# On the Sensitivity of the Virion Envelope to Lipid Peroxidation

**DOI:** 10.1128/spectrum.03009-22

**Published:** 2022-09-20

**Authors:** Consuelo B. Correa Sierra, Luis M. Schang

**Affiliations:** a Department of Microbiology and Immunology and Baker Institute for Animal Health, College of Veterinary Medicine, Cornell University, Ithaca, New York, USA; Fundacio irsiCaixa

**Keywords:** antiviral, lipoperoxidation, herpesviruses

## Abstract

Emerging viruses are a public health threat best managed with broad spectrum antivirals. Common viral structures, like capsids or virion envelopes, have been proposed as targets for broadly active antiviral drugs. For example, a number of lipoperoxidators have been proposed to preferentially affect viral infectivity by targeting metabolically inactive enveloped virions while sparing metabolically active cells. However, this presumed preferential virion sensitivity to lipoperoxidation remains untested. To test whether virions are indeed more sensitive to lipoperoxidation than are cells, we analyzed the effects of two classic generic lipoperoxidators: lipophilic 2,2′-azobis(2,4-dimethylvaleronitrile) (AMVN) and hydrophilic 2,2′-azobis(2-methylpropionamidine) dihydrochloride (AAPH) on Vero and human foreskin fibroblasts (HFF) cell viability, HSV-1 plaquing efficiency, and virion and cell lipoperoxidation. Cells or virions were incubated with the lipoperoxidators at 37°C for 2 h or incubated in atmospheric O_2_, and dose responses (half maximal cytotoxic and effective concentration [CC_50_ and EC_50_]) were evaluated by three or four parameter regression. The HSV-1 virions were slightly more sensitive to lipoperoxidators than were the cells (selectivity index [SI], 3.3 to 7.4). The effects of the lipophilic AMVN on both cell and virion viability directly correlated with the extent of membrane lipoperoxidation as evaluated by two different probes, C11-Bodipy and liperfluo. Moreover, the hydrophilic AAPH-induced virion inactivation at lower concentrations than did lipoperoxidation. Known lipoperoxidators inhibit infectivity via lipoperoxidation-independent mechanisms. Antioxidants protected against a loss of viral infectivity by less than 5-fold. Carrier bovine serum albumin (BSA) protected against both peroxidators to a similar extent when present together with the lipoperoxidating agents, suggesting that BSA quenches them as expected. Virions incubated in atmospheric oxidative conditions suffered losses of infectivity that were similar to those of chemically peroxidated virions, and they were protected by water soluble vitamin C and BSA with no evident lipoperoxidation, indicating predominant peroxidative damage to nonlipid virion components. Thus, lipoperoxidation is not a mechanism by which to specifically inhibit the infectivity of enveloped viruses, and the effects of known lipoperoxidators on virion infectivity are not solely mediated by lipoperoxidation.

**IMPORTANCE** Small molecules that induce lipoperoxidation have been proposed repeatedly as potential antiviral drugs based on a presumed unique sensitivity of virions to this type of damage. Several small molecules that inactivate virions without affecting cells have been proposed to act primarily by inducing lipoperoxidation. However, the preferential sensitivity of virions to lipoperoxidators had not been experimentally evaluated. Using two of the best characterized small molecule lipoperoxidators, which are widely considered to be the prototypical water soluble and liposoluble lipoperoxidators, we show that lipoperoxidators have no preference for virions over cells. Moreover, they also inactivate virions by mechanisms other than the induction of lipoperoxidation. Therefore, the general induction of lipoperoxidation is not a path by which to develop antivirals. Moreover, molecules with specific antiviral activity which are not cytotoxic and have no preference to localize to virions over cells are unlikely to act primarily by inducing lipoperoxidation.

## INTRODUCTION

Although about three quarters of the viruses that cause disease in humans are enveloped, only 11 of them are targeted with specific clinically approved antivirals (1), and these most often target the viral DNA or RNA polymerases or proteases (2). Only five antivirals are approved for use against more than a couple of related viruses: lamivudine, tenofovir, foscarnet, ribavirin, and valacyclovir, and the clinical and virological responses to approved antivirals by viruses other than hepatitis C virus, and perhaps SARS-CoV-2, can be suboptimal (3).

Emerging and reemerging viruses constitute hard-to-predict threats which are consequently difficult to control with antivirals that target specific viral proteins. Such antivirals can only be developed after a pathogen has been identified and characterized, which results in a significant lag time, as is exemplified by the ongoing SARS CoV-2 pandemic. Moreover, selection for resistance constitutes a challenge for direct acting antivirals and most often results in the need for combination therapy (4).

Broad spectrum antivirals could be used to treat emerging, as well as established, viral infections. The challenge to developing them lies in that they require targets necessary for the replication of many viruses, but viruses differ in genomic material, replication sites, structures, viral proteins, and requirements for cellular proteins. Although some cellular proteins are required for the replication of many viruses, an antiviral inhibiting them should result in no toxic effects to the entire organism. Consequently, no drug targeting these proteins has been developed into an antiviral (5–9), even though many cellular proteins are targeted by drugs developed for non-infectious diseases, including the cyclin-dependent kinases (CDK) (10, 11), which are involved in the replication of many unrelated viruses.

Viral proteins conserved among different viral families constitute potential targets for broad spectrum antivirals. The most commonly targeted of such proteins are the viral RNA dependent RNA polymerases (RdRP) (12). Viral DNA polymerases are also targets of effective antivirals, some of which inhibit more than one virus (13, 14).

Viruses also share some structural features. However, antivirals based on targeting common virion structures must still have no deleterious side effects. The efforts have focused largely on targeting capsids, which have no cellular counterpart. These antivirals prevent the spread of the infection by virions produced by treated cells as well as by the amplification of viral genomes, and they could also be able to prevent primary infections (15–21).

Viral envelopes have also been proposed as targets for broadly active antivirals (22–25). The viral envelope is constituted by a lipid bilayer with embedded viral glycoproteins. The glycoproteins are encoded in the viral genome and differ among different viruses, whereas the lipids are acquired from the cell during budding (26) and vary less. Moreover, the size, lipid composition, and protein percentage of the virion envelopes differ from those of the average cellular membranes (27). However, trafficking cellular vesicles have similar diameters to virions, the lipid composition of the virion envelope usually resembles that of the membrane from which they bud, and the inner mitochondrial membrane has a similarly high protein content to most virions. Thus, none of these characteristics is unique to the virion envelope.

However, there is a major difference between viruses and cells in that viruses are metabolically inert. Virions have no source of energy for fusion other than that provided by the attachment, binding, and rearrangement of their glycoproteins (28). Virions cannot modify their envelope lipid composition, either, and the lipid composition of each leaflet affects the fusion energy barrier. Increasing the fusion energy barrier thus impedes fusion and infectivity (29–31).

Hypericin, hypocrellins, and porphyrins were first described as potential antivirals more than 30 years ago (32). Their mechanism of action was initially proposed to be the lipoperoxidation of virion envelopes. Other mechanisms have been proposed since, as viral proteins and nucleic acids are also targets of the oxidative damage (33). These compounds are unsurprisingly cytotoxic (33). More recently, other compounds that target specifically enveloped viruses without cytotoxic effects have also been proposed to act via lipoperoxidation. Many of these compounds have a higher antiviral potency when exposed to light in the presence of oxygen (34). Rhodamine (35), thiobarbituric derivatives (36), phthalocyanine-based components (37), LJ001 (23), and the rigid amphipathic fusion inhibitors (RAFIs) (38) all inhibited the infectivity of several enveloped viruses. LJ001 and the RAFIs were not cytotoxic at concentrations 100-fold higher than those of the antivirals. The precise mechanism of the antiviral action of most of these compounds remains to be identified, but lipid peroxidation has been often proposed (36, 39–43).

Lipid peroxidation is a chain reaction initiated by oxidants (lipoperoxidators) attacking unsaturated lipids and then is extended as a chain reaction among unsaturated lipids by the lipid peroxyl radicals and other products generated by the primary attack. Electrons are drawn toward double bonds, leaving the bonds next to them more susceptible to attack. Lipid peroxidators extract a proton from them, causing a subsequent electron rearrangement that generates a reactive lipid radical, which then reacts with molecular oxygen (O_2_) to generate a lipid peroxyl radical. Lipid peroxyl radicals then attack nearby unsaturated lipids, forming more peroxyl radicals, hydroperoxides, and other secondary products (44, 45). Lipid peroxidation increases membrane permeability and fluidity and results in major membrane damage (46); its secondary products also cause protein and DNA damage (47, 48).

The proposed use of the lipoperoxidation of enveloped viruses as an antiviral mechanism (36, 49, 50) relies on the presumption that virions are more sensitive to any lipid peroxidator than are cells, a premise that remains untested to this day. Thus, we tested the effect of the classical chemical peroxyl radical generators AMVN or AAPH (44) on virion infectivity, cell viability, and the lipoperoxidation of virions and cells, as well as the effects on virion infectivity and lipoperoxidation under atmospheric oxygen conditions. Our results indicate that lipoperoxidation does not specifically target virions and that lipoperoxidators or oxidative conditions also reduce virion infectivity by affecting other targets besides envelope lipids.

## RESULTS

### Virions and cells are similarly sensitive to classic generic lipoperoxidators.

To test whether the induction of envelope lipid peroxidation could be used as a broad spectrum strategy against enveloped viruses, we evaluated the effect of well-characterized chemical lipoperoxidators in cell viability and virion infectivity. We selected the classical chemical lipophilic AMVN and hydrophilic AAPH peroxyl radical generators to initiate the peroxidation chain reaction from inside the lipid bilayers or the aqueous environment, respectively. We tested their effects on two different mammalian cell lines, human primary fibroblasts HFF and immortalized African green monkey Vero cells, as different cells have different susceptibilities to lipoperoxidation (51, 52).

To evaluate the effects on cell viability, subconfluent cell monolayers were incubated in a medium containing selected concentrations of AMVN, AAPH, or vehicle for 2 h at 37°C in 5% CO_2,_ and cell viability was assessed immediately after.

To evaluate the effects on virions, approximately 200 plaque-forming units (PFU) of HSV-1 in DMEM without serum were exposed to AMVN, AAPH, or vehicle for 2 h at 37°C before the inoculation semi-confluent Vero or confluent HFF cell monolayers with the pretreated virions for 1 h. The inocula were removed, and the cells were washed and then incubated at 37°C in 5% CO_2_ until plaques were visible and well-resolved (about 2 days), at which time they were fixed and stained with crystal violet.

**FIG 1:**
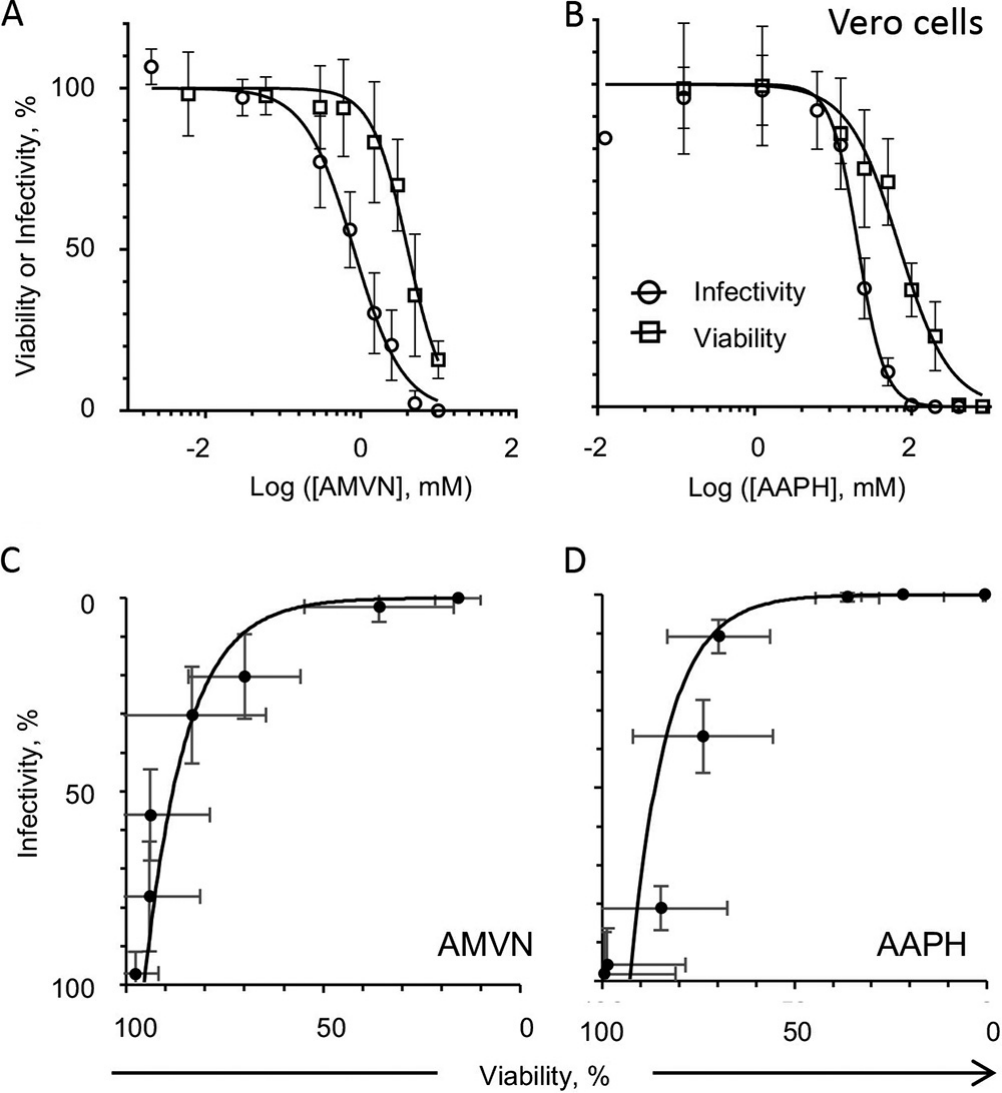
Effects of classical peroxidators on cell viability or virion infectivity to Vero cells. Liposoluble (A) or water soluble (B) peroxidator effects on virion infectivity (empty circles) or Vero viability (empty squares). Correlation analyses of virion infectivity and cell viability (black filled circles) treated or not treated with a liposoluble ([C], y = 0.0117e^0.0948×^, R^2^ = 0.97) or water soluble peroxidator ([D], y = 0.0059e^0.1048x^, R^2^ = 0.77). Average ± standard deviation (SD); *n* = 3 to 4.

As expected, the induction of lipoperoxidation by AMVN or AAPH resulted in dose-dependent responses. Increasing peroxidator concentrations resulted in increasing losses of cell viability and virion infectivity (Fig. 1A and B; Table 1). AMVN started to reduce cell viability from 0.3 mM and virion infectivity from 1.5 mM, whereas AAPH started to reduce cell viability from 12.5 mM and virion infectivity from 50 mM. The maximal inhibition of cell viability or virion infectivity was reached at 10 mM and 5 mM for AMVN or 400 mM and 100 mM for AAPH.

**TABLE 1:** Antiviral potency and cytotoxicity of liposoluble (AMVN) and water soluble (AAPH) soluble peroxidators as well as protection by antioxidants^*a*^

				Vitamin E		Vitamin C	
Antioxidant:		CC_50_/EC_50_/SI	None		PI		PI
AMVN	VERO	CC_50_^*b*^	4.0	4.6	1.2^*d*^	3.3	0.8
		EC_50_^*b*^	0.8	3.8	4.8^e^***	2.6	3.3**
		SI^*c*^	5.0**	1.2	0.2^*f*^	1.3	0.3
	HFF	CC_50_^*b*^	4.8	4.9	1.0	4.4	0.9
		EC_50_^*b*^	1.1	2.7	2.5	1.4	1.3
		SI^*c*^	4.4^c^**	1.8	0.4	3.1	0.7
AAPH	VERO	CC_50_^*b*^	69.0	135.0	2.0*	88.4	1.3
		EC_50_^*b*^	20.8	31.0	1.5	100.9	4.9
		SI^*c*^	3.3*	4.4	1.3	0.9	0.3
	HFF	CC_50_^*b*^	182.2	96.7	0.5*	193.9	1.1
		EC_50_^*b*^	24.7	31.4	1.3*	113.2	4.6***
		SI^*c*^	7.4***	3.1	0.4	1.7	0.2

a *, 0.01,  < *P* < 0.05; **, 0.001 < *P* ≤ 0.01; ***, P ≤ 0.001 (Student’s *t* test)

b mM

c CC_50_/EC_50_

d CC_50 (antioxidant)_/CC_50_

e EC_50 (antioxidant)_/EC_50_

f PI (cells)/PI (virus)

The relationship between the effects of AAPH or AMVN on cell viability or virion infectivity was exponential, with infectivity being more sensitive than viability (Fig. 1C and D). However, AMVN was only slightly more potent against virions than against cells (CC_50,_ 4 mM; EC_50,_ 0.8 mM) (Fig. 1A; Table 1), as was AAPH (CC_50_, 69 mM; EC_50_, 20.8 mM) (Fig. 1B; Table 1). The SI were only 4.9 and 3.3 for the lipid soluble or water soluble lipoperoxidators, respectively.

**FIG 2:**
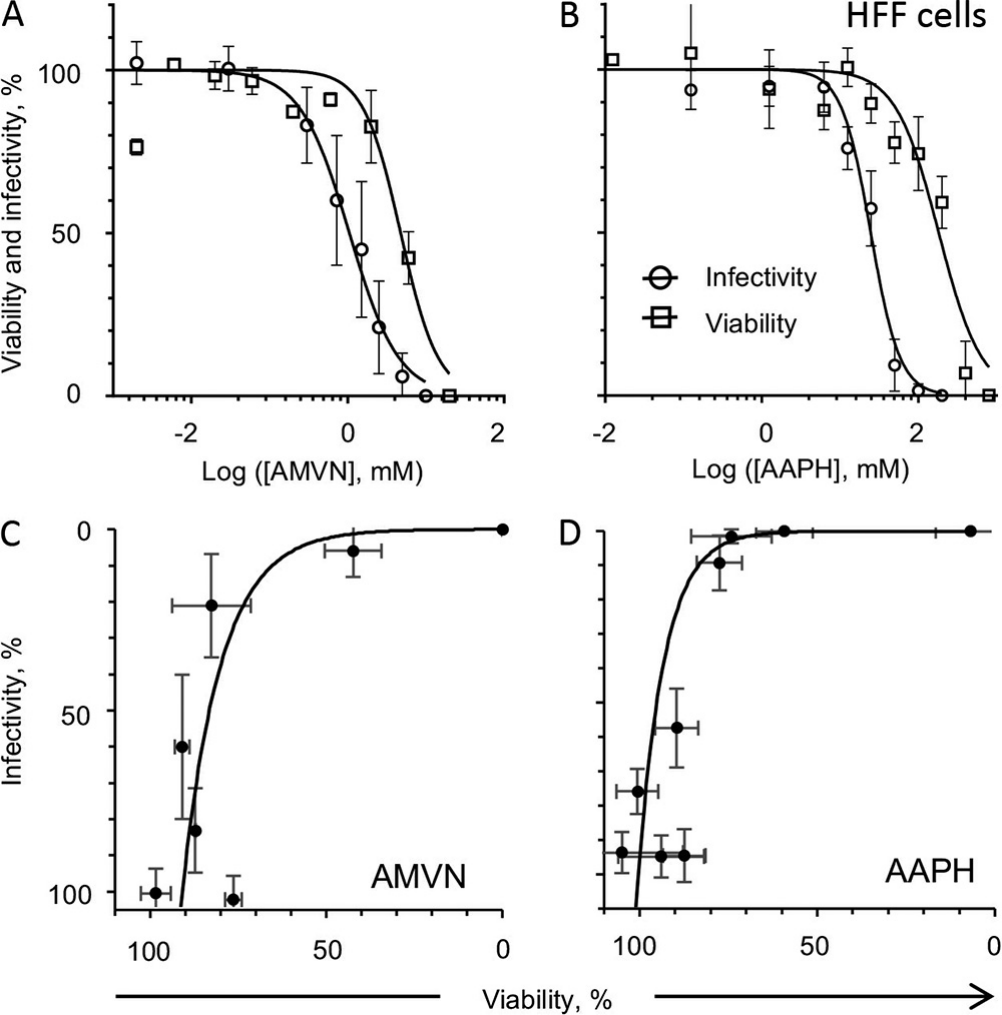
Effects of classical peroxidators on HFF viability or virion infectivity to HFF cells. Liposoluble (A) or water soluble (B) peroxidator effects on virion infectivity (empty circles) or Vero viability (empty squares). Correlation analyses of virion infectivity and cell viability (black filled circles) treated or not treated with a liposoluble ([C], y = 0.0279e^0.09x,^ R^2^ = 0.4197) or water soluble peroxidator ([D], y = 0.0001e^0.1358x^, R^2^ = 0.4059). Average ± SD; *n* = 3 to 4.

Similar results were obtained with the HFF cells (Fig. 2; Table 1), whose viability was affected from 2 mM AMVN, whereas virion infectivity was affected from 0.7 mM (Fig. 2A). HFF viability was maximally affected from 50 mM AAPH, and virion infectivity was maximally affected from 12.5 mM (Fig. 2B). The SI in HFF cells were 4.5 and 7.4 for the lipid soluble or the water soluble lipoperoxidators, respectively, and the effects on infectivity and viability were poorly correlated (Fig. 2C and D; Table 1).

The HSV-1 virions were only 3 to 7.4-fold more sensitive to lipoperoxidators than was either cell line (Table 1). Thus, we considered the possibility that the apparent antiviral activity could have resulted from the killing of the infected cells by a peroxidator carried with the pretreated infecting virions. To test for this possibility, virions were incubated with AMVN for 2 h at 37°C, diluted 1:100, and added to cells for 1 h at 37°C as before. The inocula were removed, the cells were washed, and the viability was immediately evaluated, mimicking the conditions used to test the direct exposure of the cells to the peroxidators.

**FIG 3:**
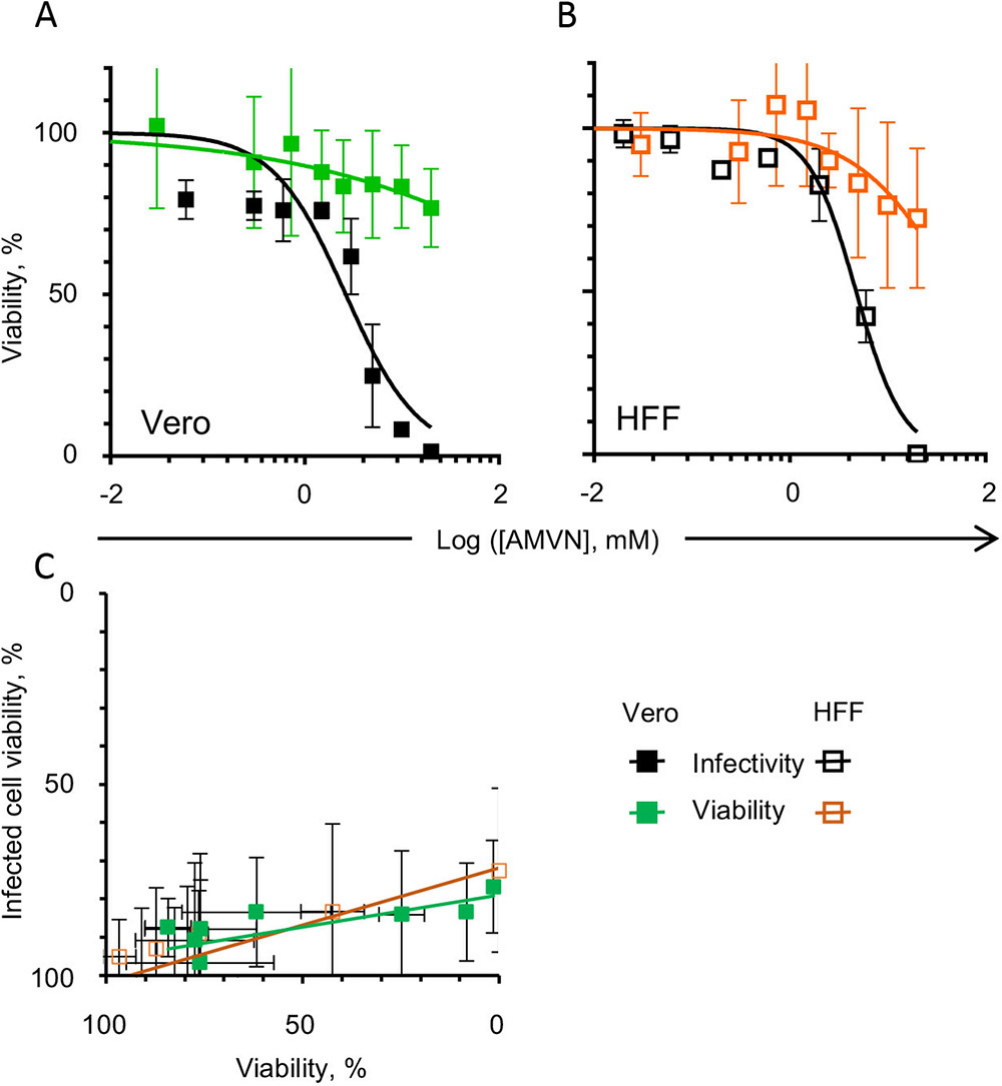
Effects of liposoluble peroxidators carried in virions on the cell viability of Vero cells (A), cell viability (black squares) direct exposure to AMVN (green squares), after exposure by virions pretreated with AMVN, or (B), HFF cells. Correlation analysis of cell viability after infection with virions pretreated with a liposoluble peroxidator with cell viability after direct exposure to a liposoluble lipoperoxidator ([C] [green squares], Vero; orange squares, HFF). Average ± SD; *n* = 3 to 4.

The viability of cells infected with treated virions was not different from that of the uninfected, untreated cells in either cell line. AMVN carried by virions had some minor effects on infected cell viability from 2 and 3 mM in the Vero and HFF cells, respectively, with cell viability decreasing by about 20 to 30% at the highest AMVN concentration in either cell line (Fig. 3A and B). The correlation between the viability of the treated cells and infected cells (Fig. 3C) had a slope of only about 15 to 30% and a large variability, indicating that any residual peroxidator carried by virions could have been responsible for just a marginal loss in cell viability, even at the highest concentration, meaning that is it unlikely to result in the observed inhibition of virion infectivity.

### Antioxidants neutralize the effects of lipoperoxidators on cells and virions.

Antioxidants act as peroxyl radical scavengers. We tested the effects of the classic water or lipid soluble antioxidants, vitamins C and E, respectively, on cell viability and virion infectivity. To test the lipid soluble antioxidant, cells or virions were preloaded with vitamin E-BSA and then treated with lipoperoxidators. To test the water soluble antioxidant, cells or virions were treated simultaneously with vitamin C and lipoperoxidators. Antioxidants are expected to have only a minor effect on cell viability, as cells have many mechanisms by which to cope with membrane lipoperoxidation. According to the model proposing that general lipid peroxidation is a specific antiviral mechanism (36, 39–43), however, antioxidants are predicted to have a large impact on viruses, which are proposed to have no mechanisms with which to deal with lipid bilayer peroxidative damage.

**FIG 4:**
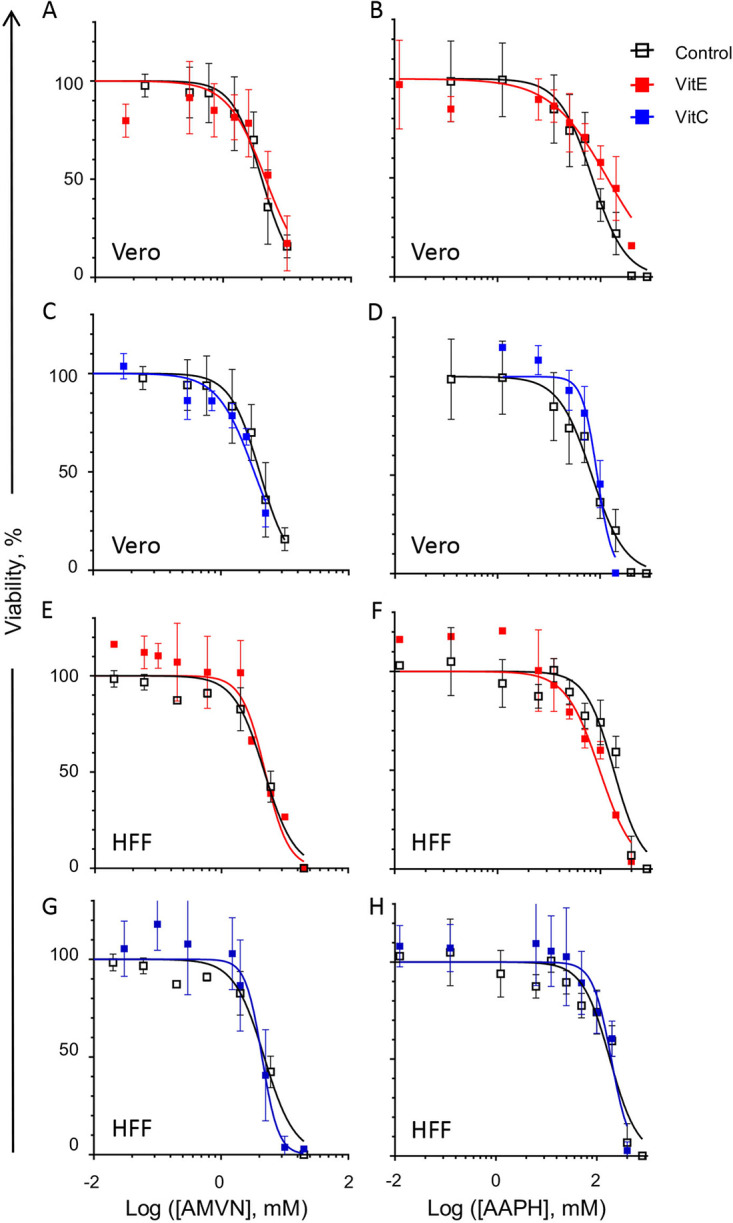
Effects of liposoluble or water soluble antioxidants (vitamin E or C, respectively) on the inhibition of the cell viability effects of classical lipid peroxidators in two cell lines. Vitamin E-BSA effects on the inhibition of cell viability by liposoluble ([A], Vero cells; [E], HFF cells); or water soluble ([B], Vero cells; (F), HFF cells) peroxidators. Vitamin C effects on the inhibition of cell viability by liposoluble ([C], Vero cells; [G], HFF cells) or water soluble ([D], Vero cells; [H], HFF cells) peroxidators. Black empty squares, cell viability without antioxidants; red squares, cell viability after preloading with vitamin E-BSA; blue squares, cell viability after treatment with vitamin C. Vitamin E uses BSA as a carrier. Average ± SD; *n* = 3.

As expected, antioxidants did not greatly affect the viability of cells treated with lipoperoxidators (0.5 to 2-fold protection) (Fig. 4; Table 1).

**FIG 5:**
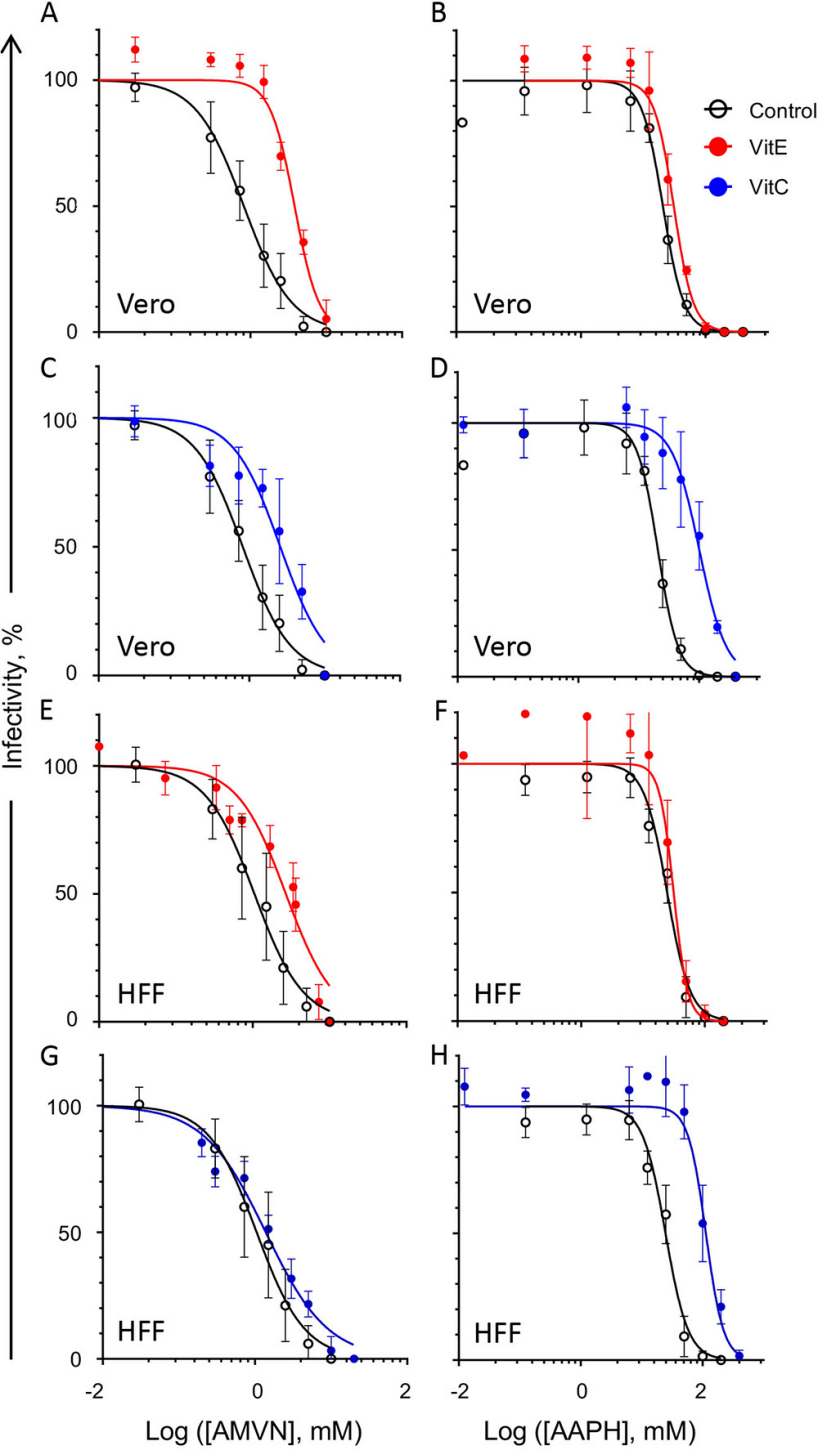
Effects of liposoluble or water soluble antioxidants (vitamin E or C, respectively) on the inhibition of virion infectivity by classical lipid peroxidators in two cell lines. Vitamin E-BSA effects on the inhibition of virion infectivity by liposoluble ([A], Vero cells; [E], HFF cells) or water soluble ([B], Vero cells; [F], HFF cells) peroxidators. Vitamin C effects on the inhibition of virion infectivity by liposoluble ([C], Vero cells; [G], HFF cells) or water soluble ([D], Vero cells; [H], HFF cells) soluble peroxidators. Black empty circles, virion infectivity without antioxidants; red circles, virion infectivity after preloading with vitamin E-BSA; blue circles, virion infectivity after treatment with vitamin C. Vitamin E uses BSA as a carrier. Average ± SD; *n* = 3.

**FIG 6:**
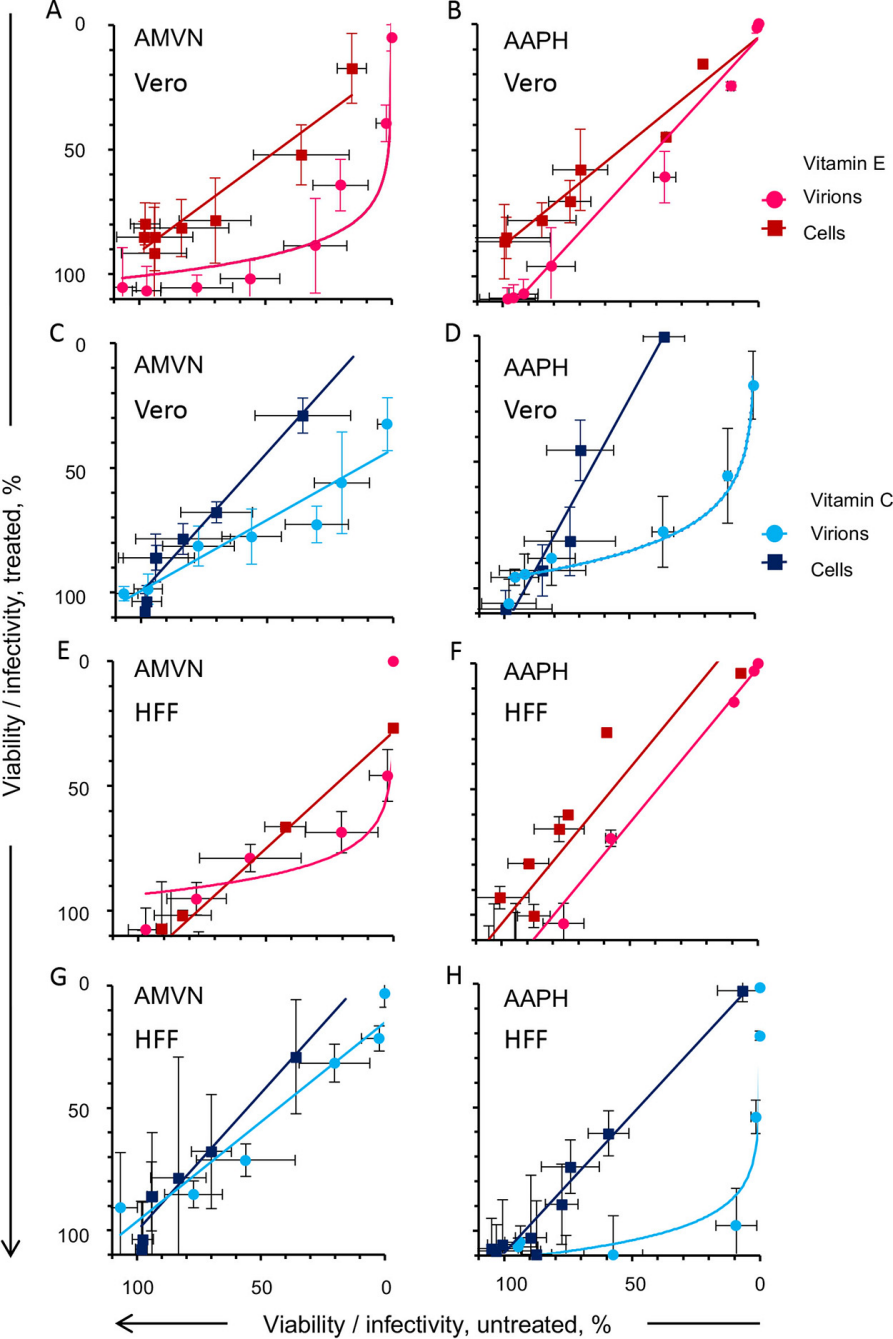
Correlation analyses of the effects of liposoluble or water soluble antioxidants (vitamin E or C, respectively) on the inhibition of cell viability or virion infectivity by classical lipid peroxidators in two cell lines. Correlation of vitamin E-BSA effects on the inhibition of cell viability or virion infectivity by liposoluble ([A], Vero cells; [E], HFF cells) or water soluble ([B], Vero cells; [F], HFF cells) peroxidators. Correlation of vitamin C effects on the inhibition of cell viability or virion infectivity by liposoluble ([C], Vero cells; [G], HFF cells) or water soluble ([D], Vero cells; [H], HFF cells) peroxidators. Red dark squares, correlation between cell viability after preloading with vitamin E-BSA versus viability without antioxidants; blue dark squares, correlation between cell viability after treatment with vitamin C versus viability without antioxidants; pink light circles, correlation between virion infectivity after preloading with vitamin E-BSA versus infectivity without antioxidants; blue light circle, correlation between virion infectivity after treatment with vitamin C versus infectivity without antioxidants. Vitamin E uses BSA as a carrier.

HSV-1 was treated with antioxidants and lipoperoxidators similarly to the cells (i.e., in DMEM without serum). In the presence of the antioxidants, the lowest effective concentrations of AMVN in the Vero cells increased from 0.3 to 1.5 mM and that of the AAPH increased from 12.5 to 25 mM (Fig. 5A to D). Similar results were obtained in HFF (Fig. 5E to H). The minimum inhibitory concentration (MIC) of AAPH that decreased infectivity in the presence of vitamin C was 100 mM in HFF and 50 mM in Vero. The lipid soluble antioxidant vitamin E protected HSV-1 against the lipid soluble peroxidator AMVN by 2.6 to 4.7-fold in the HFF and Vero cells, respectively (Table 1). Likewise, the water soluble antioxidant vitamin C protected HSV-1 against the water soluble peroxidator AAPH by 4.6 or 4.9-fold in HFF and Vero, respectively. Vitamin E, however, did not protect against the water soluble peroxidator (Fig. 5B and F; Table 1), suggesting that inactivation proceeds mostly through the peroxidation of virion components other than lipids. The protection of HSV-1 infectivity against either peroxidator was less than 5-fold. Fitting a linear regression, the toxicity of the lipoperoxidators for both cell lines was highly correlated when the cells were treated with or without antioxidants (R^2^ ≥ 0.8) (Fig. 6). The correlation between virion infectivity after preloading or not with vitamin E and treatment with the lipid soluble peroxidator was logarithmic (Fig. 6A and E). The same was observed for the relation between the treatment of the water soluble antioxidant and the water soluble peroxidator (Fig. 6D and H), indicating protection. The correlation between virion infectivity when treated or not with vitamin E and the water soluble peroxidator was linear (Fig. 6B and F), as was that between the virions treated or not with vitamin C and the lipid soluble peroxidator (Fig. 6C and G). Thus, virion infectivity is partially protected by antioxidants in the same phase as the lipoperoxidator but not when the antioxidant and peroxidator are in different phases. This result is consistent with different mechanisms of virion inactivation by water soluble or lipid soluble peroxidators. The correlation of the viability of cells preloaded with the lipid soluble antioxidant vitamin E-BSA or with the water soluble antioxidant and treated with the lipoperoxidators indicates minimal to no protection, as expected; all of the correlations fit a linear one-to-one regression (Fig. 6).

**FIG 7:**
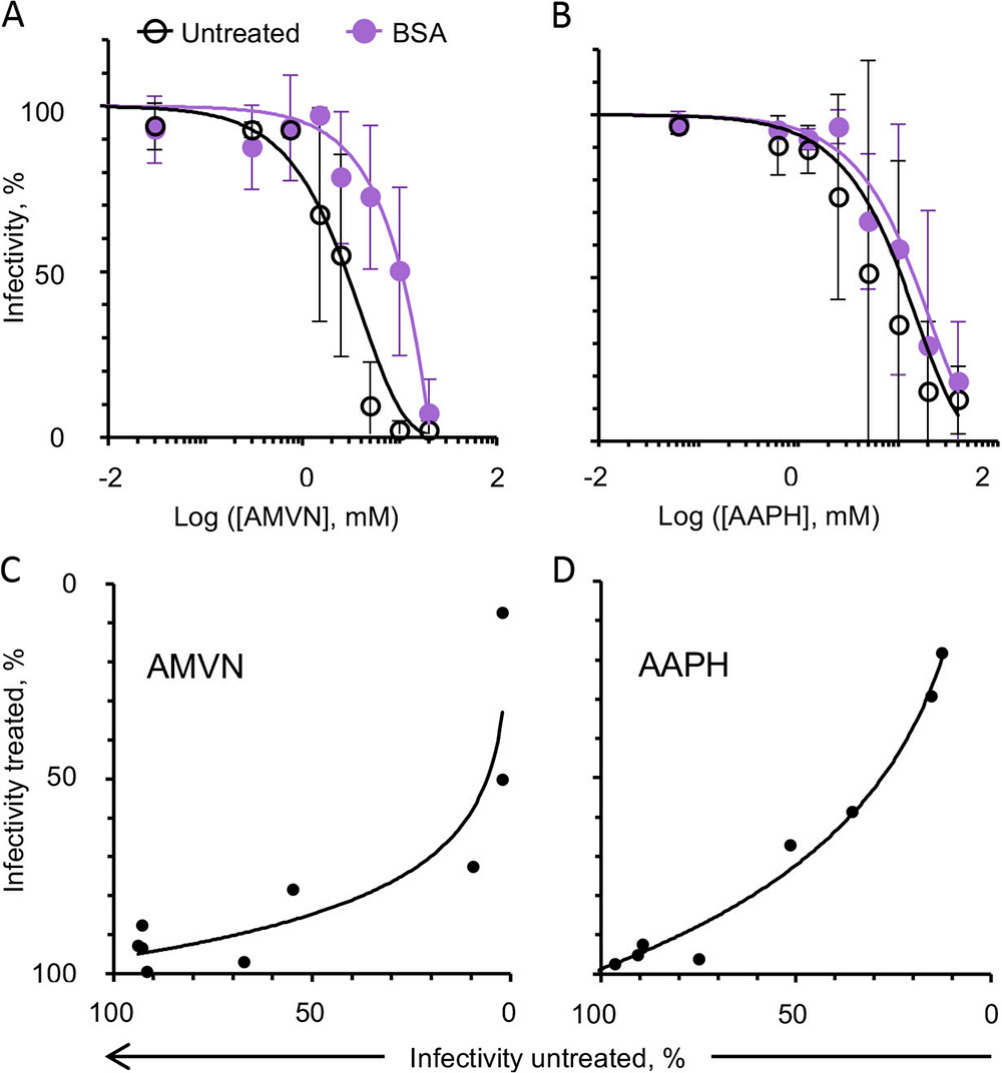
Effect of carrier BSA on the inhibition of virion infectivity by classical lipid peroxidators to Vero cells. BSA effects on the inhibition of virion infectivity by liposoluble (A) or water soluble (B) peroxidators. Correlation analyses of BSA effects on inhibition of virion infectivity by liposoluble ([C], y = 16.247ln[x] + 21.189, R^2^ = 0.807) or water soluble ([D], y = 38.177ln[x] - 77.118, R^2^ = 0.9835) peroxidators. Black empty circles, virion infectivity without antioxidants; purple circles, virion infectivity after BSA treatment; black filled circles, correlation between virion infectivity after treatment with BSA and virion infectivity without antioxidant. Average ± range; *n* = 2.

As vitamin E is insoluble in water, it is presented to cells or virions in a BSA carrier. To analyze the potential effects of any residual carrier BSA itself, HSV-1 was simultaneously treated with BSA and the lipoperoxidators for 2 h at 37°C. Treated virions were then diluted, and plaquing efficiency assays were performed. The carrier BSA appears to have protected virions against AAPH and AMVN, increasing the EC_50_ by 2- or 4-fold, respectively (Fig. 7A and B). Virion infectivity decreased starting from 2.5 mM for AMVN or 50 mM for AAPH in the presence of BSA in comparison to 0.3 or 12.5 mM in its absence, respectively. There was a high correlation between the infectivity with and without BSA treatment (Fig. 7C and D). The correlations fit logarithmic regressions, indicating that BSA apparently protects against both peroxidators when BSA and the peroxidating agent are present together, most likely by quenching them.

### Classical peroxidators induce the expected lipoperoxidation of virions and cells.

The effects of peroxidators have been commonly evaluated against purified lipids and liposomes as a proxy, but they have not been evaluated against virions (50, 53–55). We instead directly evaluated the extent of virion lipid peroxidation. To this end, cells or virions were preloaded with liperfluo or with C11-Bodipy for 30 min at 37°C before treating them with AMVN or AAPH for 2 h in DMEM without serum.

**FIG 8:**
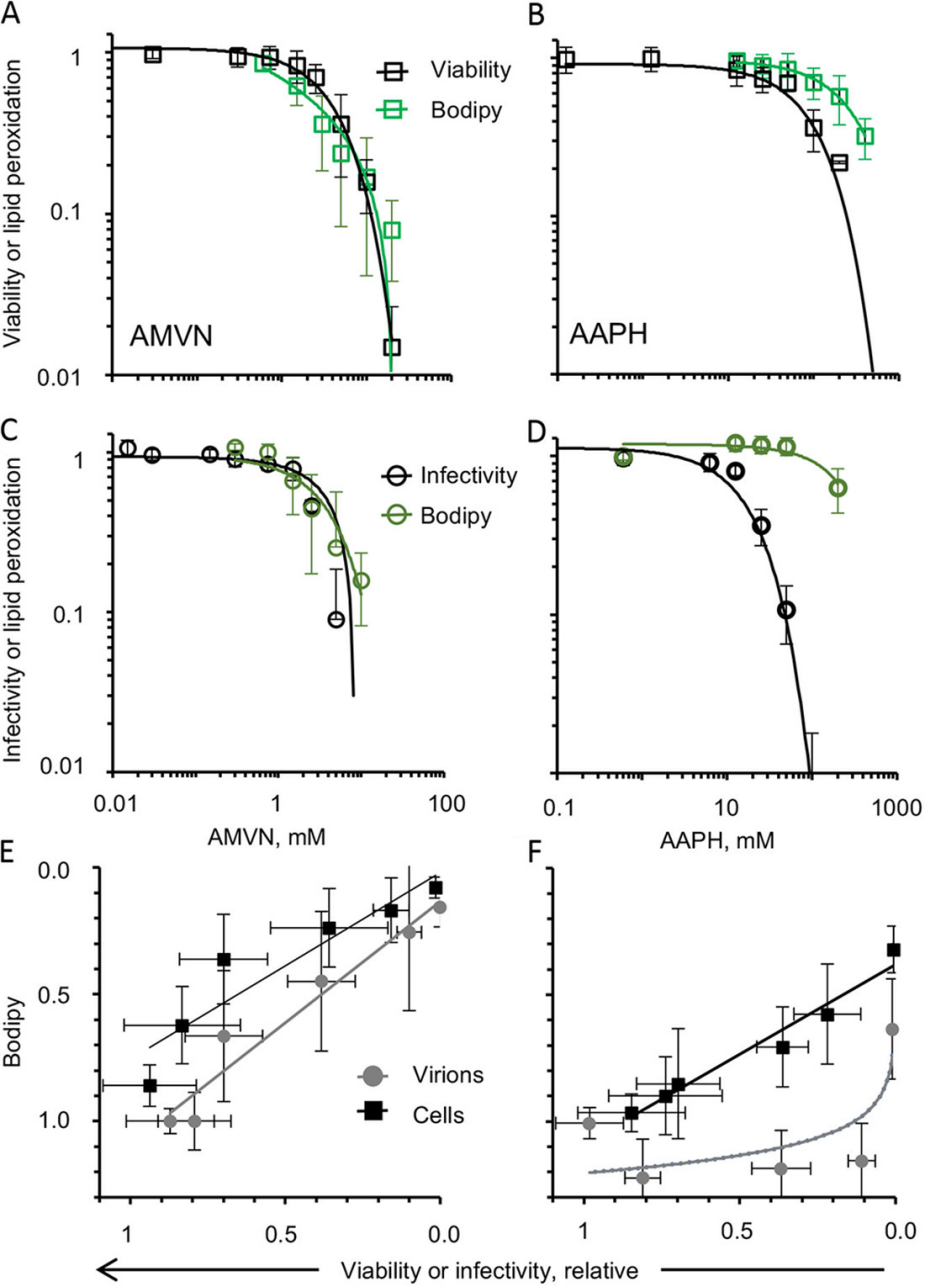
Effects of general peroxidators on cell viability, virion infectivity, or lipid peroxidation and their correlation. Liposoluble peroxidator effects on cell viability and lipoperoxidation of Vero cells (A) or virion infectivity and lipoperoxidation of virions (C). Water soluble peroxidator effects on cell viability and lipoperoxidation of Vero cells (B) or infectivity and lipoperoxidation of virions (D). Correlation analyses of the effects of classical lipoperoxidators on cell viability, virion infectivity, and lipoperoxidation (liposoluble [E] or water soluble [F] peroxidators). Black empty squares, cell viability; green squares, lipoperoxidation of cell membranes; black empty circles, virion infectivity; green circles, lipoperoxidation of virion envelopes; black filled squares, correlation between cell viability versus lipid peroxidation of cell membranes; gray filled circles, correlation between virion infectivity versus lipid peroxidation of virion envelope. Lipoperoxidation was evaluated by C11-Bodipy. Average ± SD; *n* = 3 to 5.

The lipid soluble AMVN affected viability or infectivity and lipid peroxidation with equivalent potencies. The lowest AMVN concentration affecting virion infectivity or virion peroxidation was between 1.5 and 2.5 mM (Fig. 8A and C). Similarly, the lowest concentration of AMVN that affected viability and cell lipid peroxidation was 2.5 mM. The decreases in infectivity or viability and the increases in peroxidation induced by AMVN were highly correlated (>0.8) (Fig. 8E) and fit a linear regression, indicating that the losses of cell viability and virion infectivity were both largely caused by lipoperoxidation.

In contrast, the water soluble peroxidator AAPH affected cell viability and virion infectivity from lower concentrations than those at which it induced detectable lipoperoxidation. AAPH was 2 to 8- fold more potent against cell viability or virion infectivity than was lipoperoxidation, respectively, indicating that it also affects viability and infectivity by some mechanisms independent of lipoperoxidation (Fig. 8B and D; Tables 1 and 2).

**TABLE 2:** Lipoperoxidation produced by liposoluble (AMVN) and water soluble (AAPH) peroxidators in virions and cells as well as protection by antioxidants^*a*^

			Bodipy					Liperfluo				
	Probe:			Vitamin E		Vitamin C			Vitamin E		Vitamin C	
	Antioxidant	CC_50_/EC_50_/SI	None		LPP		LPP	None		LPP		LPP
Lipoperoxidator	AMVN	CP_50_^*b*^	2.1	3.5	1.7^*d*^	1.7	0.8	12.6	16.1	1.3	11.1	0.7
		VP_50_^*b*^	2.7	2.4	0.9^*e*^	2.0	0.7	1.2	0.9	0.8	1.2	1.3
		LPI^*c*^	0.8	1.5	1.9^*f*^	0.9	1.1	10.5	17.9	1.7	9.3	0.5
											
	AAPH	CP_50_^*b*^	238	202	0.8	528	2.2	89.2	90.7	1.0	90.7	1.0
		VP_50_^*b*^	>400	>400	NA	>400	NA	229.9	238	1.0	222.9	1.0
		LPI^*c*^	NA	NA	NA	NA	NA	0.4	0.4	1.0	0.4	1.0

a CP_50_ Cell peroxidation_50_, concentration of peroxidant that results in 50% peroxidation in cell lipids; VP_50_ Virion peroxidation_50_, concentration of peroxidant that results in 50% peroxidation in virion lipids; LPI, lipoperoxidation selectivity index; LPP, lipoperoxidation protection factor; >400, larger than the highest tested concentration; NA, not available (primary parameters in these derivative values could not be measured).

b mM

c CP_50_/VP_50_

d CP_50 (antioxidant)_/CP_50_

e VP_50 (antioxidant)_/VP_50_

f LPI_(virus)_/LPI_(cell)_

The lowest AAPH concentration that decreased cell viability or virion infectivity was 25 mM, while the minimum concentration that increased peroxidation was 50 to 100 mM for cells or 200 mM for virions (Fig. 8B and D). AAPH induced lipoperoxidation and loss of cell viability were highly correlated and fit a linear regression, while AAPH induced lipoperoxidation and loss of virion infectivity did not correlate, as it only induced detectable levels of virion lipoperoxidation at the highest concentrations (Fig. 8F).

**FIG 9:**
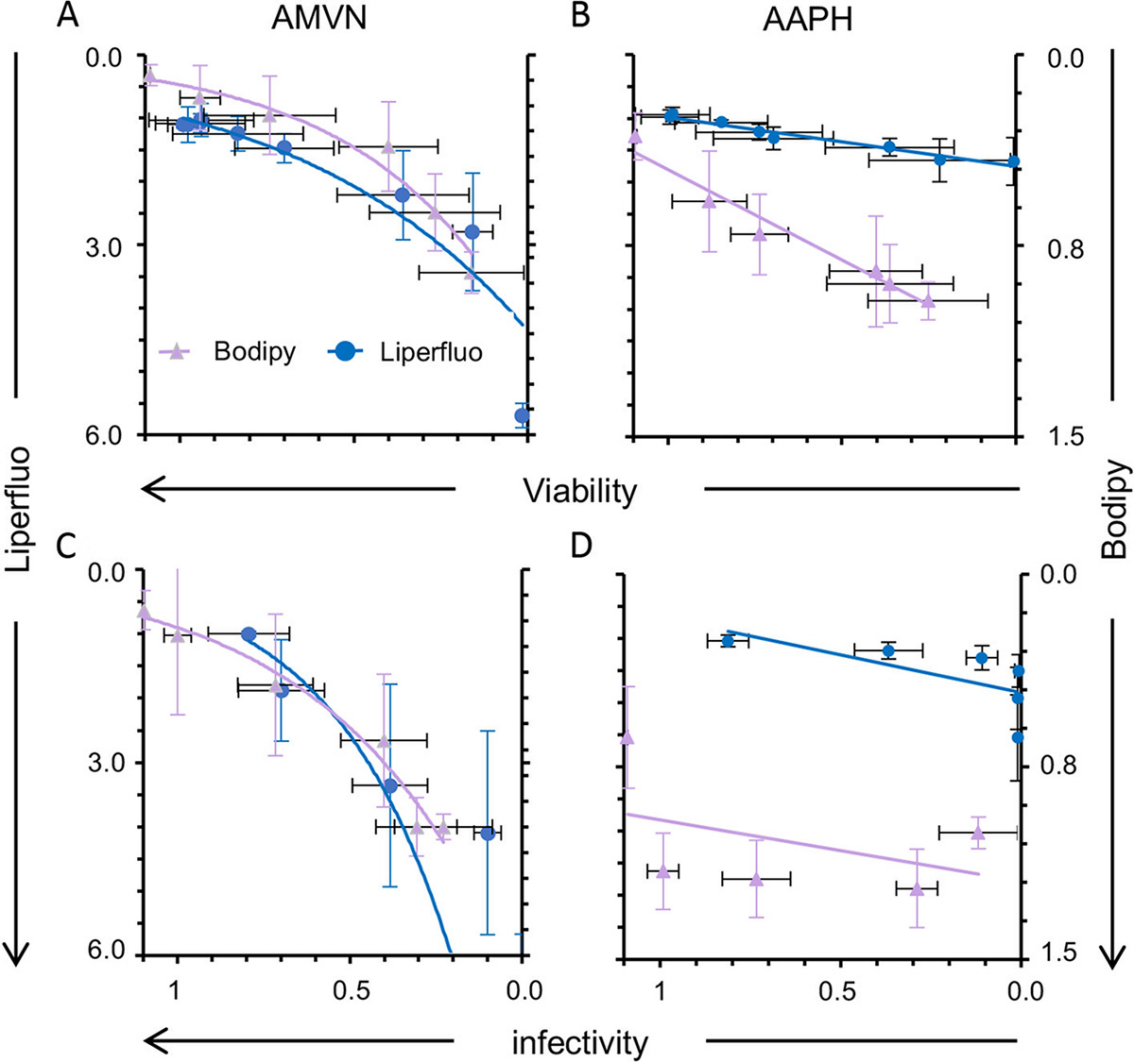
Correlation analyses of the effects of classical liposoluble peroxidators on cell viability or virion infectivity and cell membrane or virion envelope lipid peroxidation. Liposoluble peroxidator effect on cell viability and lipoperoxidation (A) and on virion infectivity and envelope peroxidation (C) evaluated with liperfluo or C11-Bodipy. Water soluble peroxidator effects on cell viability and lipoperoxidation (B) and on virion infectivity and envelope peroxidation (D) evaluated with liperfluo (blue circles) or C11-Bodipy (light violet triangles). One data point is out of scale in (C).

The levels of AMVN-induced lipid peroxidation of cells or virions were consistent when evaluated by liperfluo or C11-Bodipy (Fig. 9A and C; Table 2). C11-Bodipy was more sensitive than liperfluo to the effects of AAPH (Fig. 9D). However, as already discussed, infectivity decreased with increasing concentrations of AAPH, but lipoperoxidation did not increase accordingly (Tables 1 and 2), regardless of the probe used.

### Effects of antioxidants on virion and cell lipoperoxidation.

To evaluate whether the protective effects of antioxidants on viability and infectivity resulted from protection against lipoperoxidation, cells and virions were preloaded with vitamin E-BSA for 3 h at 37°C and then with liperfluo or C11-Bodipy for 30 min at 37°C. AMVN or AAPH were added afterwards, and the virions or cells were then incubated for 2 h at 37°C. To test the effect of a water soluble antioxidant, virions or cells preloaded with liperfluo or C11-Bodipy were treated together with 10 μM vitamin C and AMVN or AAPH for 2 h at 37°C before evaluating fluorescence.

**FIG 10:**
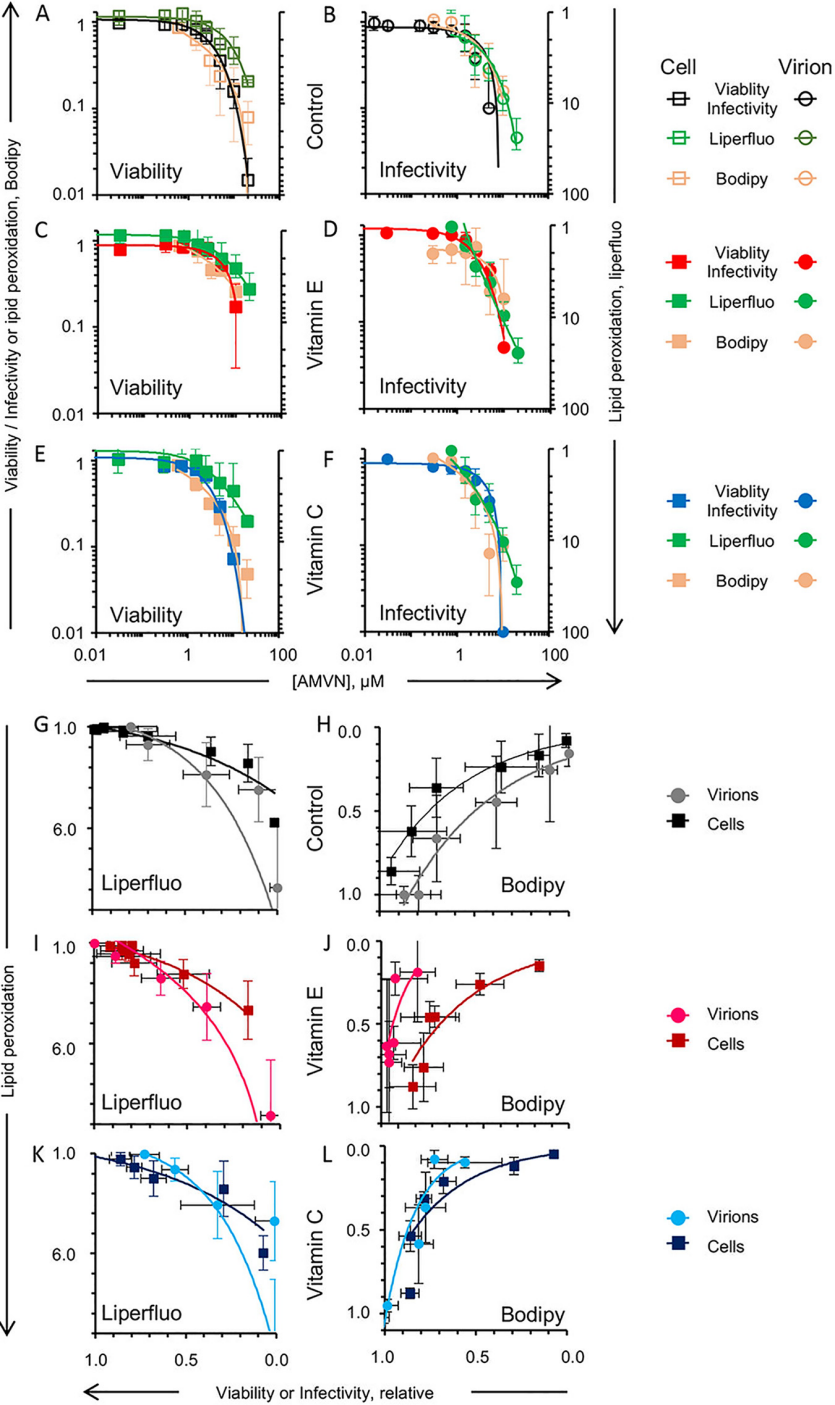
Effects of liposoluble or water soluble antioxidants (vitamin E or C, respectively) on the inhibition of the lipid peroxidation of cells or virions by the liposoluble peroxidator AMVN and their correlation with cell viability or virion infectivity. Antioxidant effects on cell lipoperoxidation and viability (A) as well as virions and virion infectivity (B) by the liposoluble peroxidator. The effects of vitamin E-BSA on cell lipoperoxidation and viability (C) as well as virions and virion infectivity (D) by the liposoluble peroxidator. The effects of vitamin C on cell lipoperoxidation and viability (E) as well as virions and virion infectivity (F) by the liposoluble peroxidator. Lipoperoxidation was evaluated by C11-Bodipy and liperfluo. Correlation analyses of the effects of liposoluble or water soluble antioxidants (vitamin E or C, respectively) on cell viability or virion infectivity and lipoperoxidation (vitamin E, evaluated with liperfluo [I] or C11-Bodipy [J]; vitamin C, evaluated with liperfluo [K] or C11-Bodipy [L]) in the absence of antioxidants (evaluated with liperfluo [G] or C11-Bodipy [H]) produced by the liposoluble peroxidator. Black empty squares, cell viability; green (liperfluo) and orange (C11-Bodipy) squares, lipoperoxidation of cell membranes; black empty circles, virion infectivity; green (liperfluo) and orange (C11-Bodipy) circles, lipoperoxidation of virion envelopes; black filled squares, correlation between cell viability versus lipid peroxidation of cell membranes; gray filled circles, correlation between virion infectivity versus lipid peroxidation of virion envelopes; red dark filled (vitamin E-BSA) and dark blue (vitamin C) squares, correlation between cell viability versus lipid peroxidation of cell membranes after preloading with antioxidants; pink light filled (vitamin E-BSA) and light blue (vitamin C) circles, correlation between virion infectivity versus lipid peroxidation of virion envelopes after preloading with antioxidants. Vitamin E uses BSA as a carrier. Average ± SD; *n* = 3 to 5.

The antioxidants had equivalent potencies when evaluated against cell viability, virion infectivity, or the respective cell or virion lipoperoxidation triggered by the liposoluble peroxidator AMVN (Fig. 10A to F; Table 2). Cell viability, virion infectivity, and lipid peroxidation with and without antioxidants were highly correlated (0.8 to 0.9 for C11-Bodipy and −0.8 to −1.0 for liperfluo) (Fig. 10G to L). The correlation between cell viability or infectivity and lipoperoxidation fit exponential regressions. Cell viability and lipoperoxidation were highly correlated (R^2^ > 0.75) (Fig. 10G to L), and so were virion infectivity and lipoperoxidation as evaluated with C11-Bodipy (R^2^ ≥ 0.7) (Fig. 10H, J, and L), but not if evaluated with liperfluo (0.4 ≤ R^2^ ≤ 0.8) (Fig. 10G, H, and K). The lowest concentration of AMVN that affected cell viability or virion infectivity was 2.5 or 1.5 mM, respectively, regardless of treatment with antioxidants. The lipoperoxidation of virions preloaded with vitamin E-BSA could not be assessed by C11-Bodipy labeling (Fig. 10J) because of the high variability observed, which likely resulted from interference.

**FIG 11:**
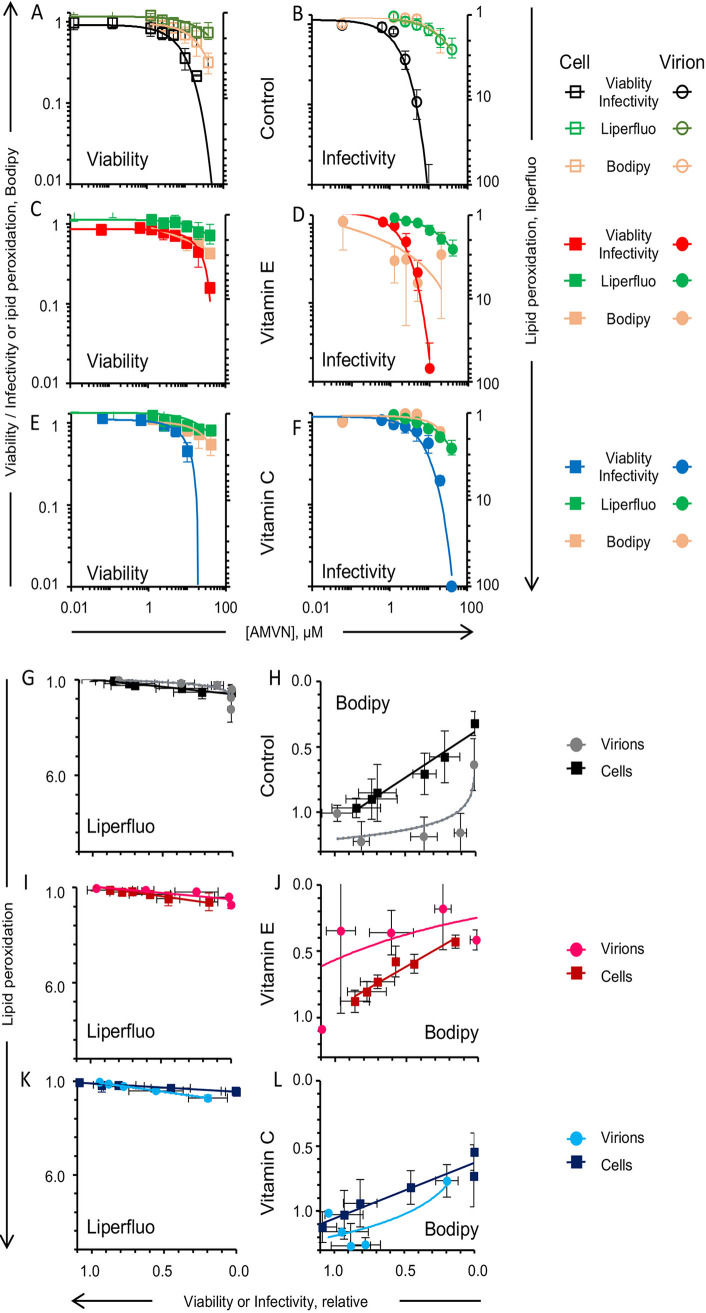
Effects of liposoluble or water soluble antioxidants (vitamin E or C, respectively) on the inhibition of the lipid peroxidation of cells and virions by the water soluble peroxidator AAPH as well as analyses of correlation with cell viability and virion infectivity. Antioxidant effects of cell lipoperoxidation and viability (A) as well as virion lipoperoxidation and infectivity (B) by the water soluble peroxidator AAPH. Vitamin E-BSA effect of cell lipoperoxidation and viability (C) and virion lipoperoxidation and infectivity (D) by the water soluble peroxidator AAPH. The effects of vitamin C on cell lipoperoxidation and viability (E) as well as virion lipoperoxidation and infectivity (F) by the water soluble peroxidator AAPH. Lipoperoxidation was evaluated by C11-Bodipy or by liperfluo. Average ± SD; *n* = 3 to 5. Correlation analyses of the effects of liposoluble or water soluble antioxidants (vitamin E or C, respectively) on cell viability or virion infectivity and their lipoperoxidation. Vitamin E, evaluated with liperfluo (I) or C11-Bodipy (J); vitamin C, evaluated with liperfluo (K) or C11-Bodipy (L) in the absence of antioxidants (evaluated with liperfluo [G] or C11-Bodipy [H]) produced by the water soluble peroxidator. Black empty squares, cell viability; green squares, liperfluo, or orange squares, C11-Bodipy, lipoperoxidation of cell membranes; black empty circles, virion infectivity; green circles, liperfluo, or orange circles, C11-Bodipy, lipoperoxidation of virion envelopes; black filled squares, correlation between cell viability versus lipid peroxidation of cell membranes; gray filled circles, correlation between virion infectivity versus lipid peroxidation of virions; red dark filled squares, vitamin E-BSA; dark blue squares, vitamin C, correlation between cell viability versus lipid peroxidation on cell membranes after preloading with antioxidants; pink light filled circles, vitamin E-BSA; and light blue circles, vitamin C, correlation between virion infectivity versus lipid peroxidation of virion envelopes after preloading with antioxidants. Vitamin E uses BSA as a carrier. Average ± SD; *n* = 3.

The preloading of antioxidants did not greatly affect the lipoperoxidation of cells or virions treated with the water soluble AAPH (Fig. 11A to F; Table 2). Preloading virions with vitamin E and C11-Bodipy before the water soluble peroxidator AAPH treatment resulted in sufficient variability so as to preclude the collection of any meaningful information (Fig. 11D). Cell viability and the lipoperoxidation of cells preloaded with antioxidants were highly correlated (1.0 for C11-Bodipy and −1.0 for liperfluo via linear regression). There was limited to no correlation between virion infectivity and the lipoperoxidation of virions preloaded with antioxidants, regardless of the probe used to evaluate the lipid peroxidation (Fig. 11G to L). The levels of lipoperoxidation detected by liperfluo in virions or cells treated with AAPH were much lower than the levels of lipoperoxidation detected with the same probe in AMVN-treated virions or cells (<2 AU at higher AAPH concentrations) (Fig. 10G, I, and K; Fig. 11G, I, and K).

### Atmospheric oxidative conditions inhibit virion infectivity but do not detectably affect virion lipoperoxidation.

**FIG 12:**
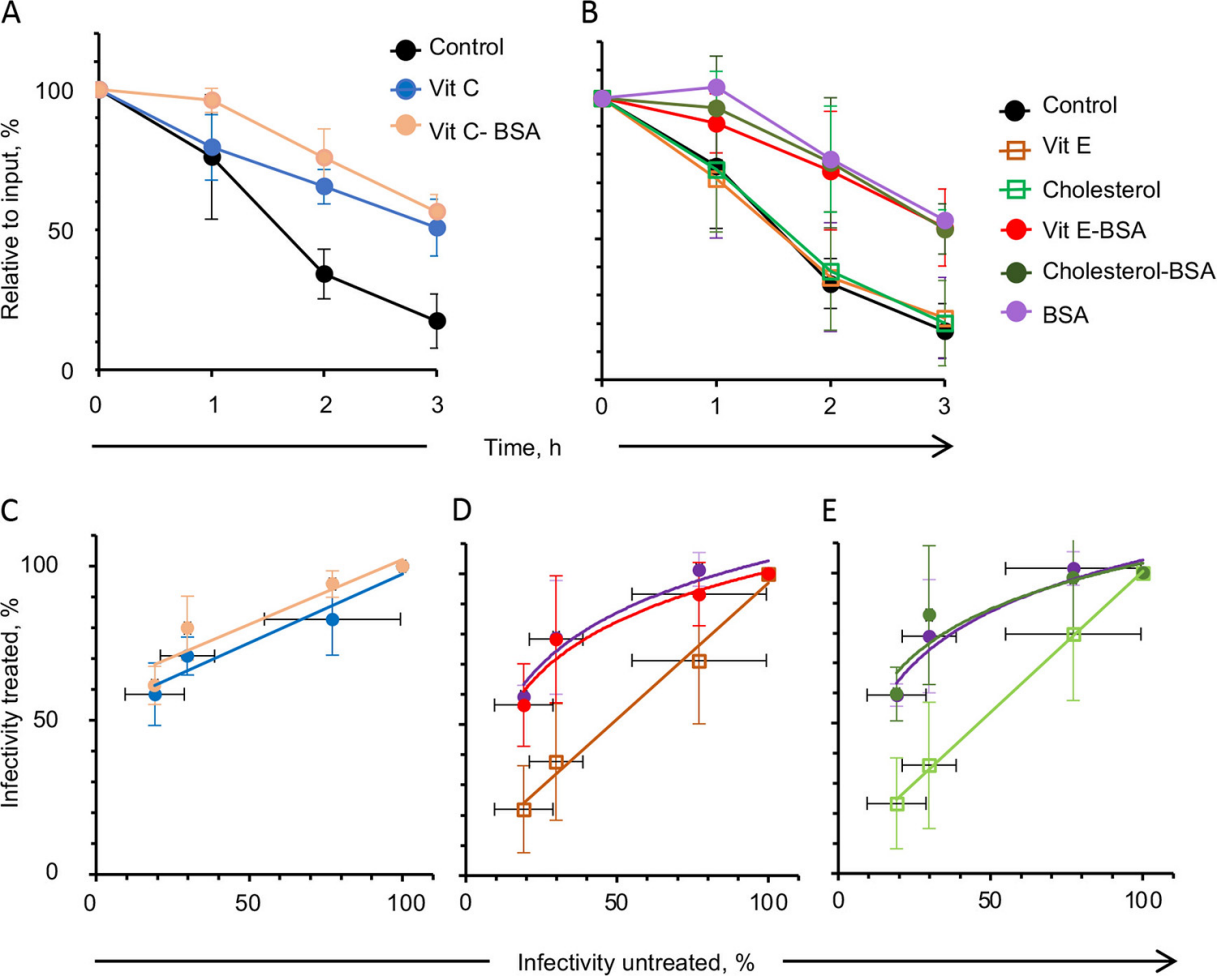
Effects of antioxidants or other molecules on the infectivity of HSV-1 incubated under oxidative conditions (atmospheric O_2_). (A) Plaquing efficiency of HSV-1 incubated in atmospheric O_2_ for up to 3 h without (black circles) or with (blue circles) vitamin C, or with vitamin C-BSA (orange circles) as well as their correlation (C). Plaquing efficiency of HSV-1 incubated in atmospheric O_2_ for up to 3 h with vitamin E (orange empty squares), cholesterol (green empty squares), vitamin E-BSA (red circles), BSA (purple circles), or cholesterol-BSA (green circles) (B) as well as their correlation (D, E). Average ± SD; *n* = 3.

To test other oxidative conditions, an HSV-1 suspension was incubated in atmospheric oxygen at 37°C in DMEM without serum. The virions were incubated with the previously used antioxidants (vitamin E-BSA and vitamin C), vitamin E without the carrier, the BSA carrier alone, vitamin C-BSA, cholesterol (another compound with a conjugated ring and alkyl chains but with limited antioxidant activity), or cholesterol-BSA. A plaquing efficiency assay was then performed with the treated virions. Viral titers dropped by about 30% after 1 h of incubation in atmospheric oxygen, a similar drop to that observed after 2 h of incubation with vitamin E-BSA, cholesterol-BSA, BSA alone, or vitamin C, whereas vitamin E or cholesterol alone had no protective effects. Carrier BSA and cholesterol-BSA protected virion infectivity by about 30% after 3h of incubation (Fig. 12A and B). A similar loss of infectivity (about 30%) was obtained with the classical lipoperoxidators after incubation for 2 h at 37°C with 0.3 or 0.7 mM AMVN or with 25 or 12.5 mM AAPH, respectively, in Vero or HFF cells. The infectivity of vitamin E or cholesterol preloaded virions was highly correlated with that of the untreated virions (>0.9) (Fig. 12D and E), and fitting linear regressions indicated no protection. The infectivity of vitamin E-BSA, cholesterol-BSA, BSA, or vitamin C preloaded virions was correlated with that of the untreated virions (Fig. 12C, D and E), and fitting logarithmic regressions indicated protection.

**FIG 13:**
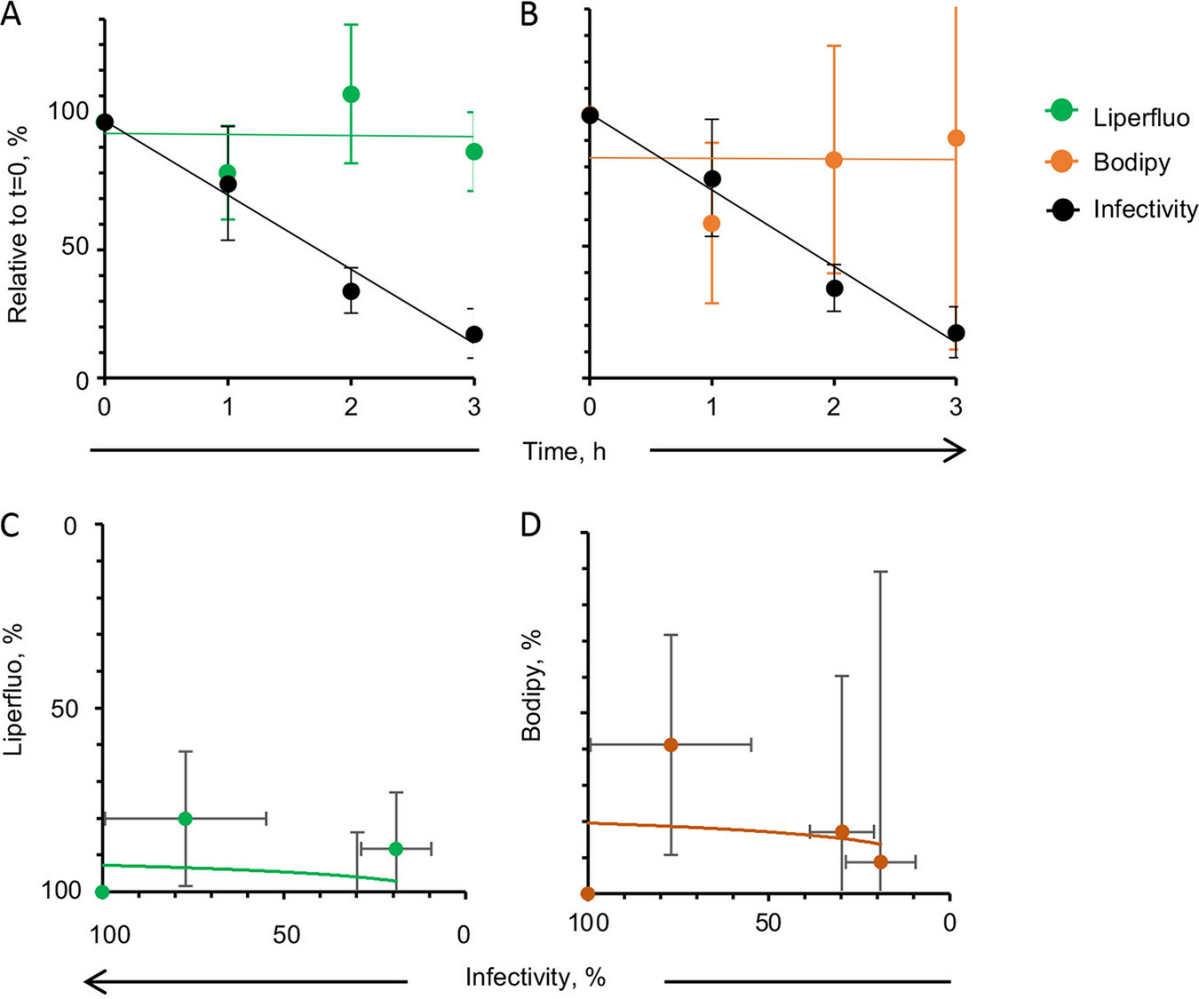
Lipid peroxidation and plaque efficiency (black circles) of HSV-1 incubated under atmospheric oxidative conditions (atmospheric O_2_) evaluated by liperfluo ([A], green circles) or C11-Bodipy (B, orange circles) as well as the correlation between peroxidation and infectivity (C, D). Average ± SD; *n* = 3.

No significant lipoperoxidation was detected in virions incubated in atmospheric O_2_ conditions for up to 3 h (Fig. 13), indicating that the losses in infectivity were largely not the result of lipoperoxidation.

## DISCUSSION

The results presented here show that lipid peroxidation does not preferentially affect virion envelope lipids over those in cellular membranes, as indicated by the narrow SI of well-characterized water soluble or liposoluble classical lipoperoxidators evaluated on two different cell lines (from 3 to 7.4) (Table 1) and by the lack of differences in lipoperoxidation potency against cells or viruses (Table 2). Moreover, antioxidants conferred only marginal protection of virion infectivity (less than 5-fold), even when the lipoperoxidator and antioxidant were in the same phase, and this protection against infectivity was not a result of protection against lipoperoxidation (Tables 1 and 2). There were no differences in the SI between the used cell lines, although their susceptibility to lipoperoxidation differed, with HFF being about 20% to 40% more sensitive. We also show that peroxidation inactivates virions by targeting components other than the lipid envelope. As expected for a water soluble peroxidator, for example, AAPH inhibited infectivity by acting on nonlipid components such that infectivity was affected at lower concentrations than those needed to induce lipoperoxidation. Another oxidative condition, atmospheric O_2,_ also resulted in a loss of infectivity that was preventable by water soluble antioxidants but not by lipid soluble ones and was not associated with envelope lipid peroxidation.

AMVN and AAPH have been widely used as lipid peroxidators for more than 30 years (56), extending to the point that antioxidant capacity is frequently tested using them. AMVN generates peroxyl radicals exclusively within the hydrophobic environment of membranes (57); in contrast, AAPH generates peroxyl radicals outside the membrane, and these radicals then diffuse into the membrane to trigger lipoperoxidation (57, 58) and induce the peroxidation of proteins or nucleic acids. These two compounds induce lipoperoxidation at a constant stable rate and were thus selected to test the model that lipid peroxidators specifically damage virion envelopes over cell membranes (59–62).

Many compounds have been proposed to inactivate virions by inducing physical damage to their envelopes through lipoperoxidation (33, 49, 63, 64). Photoactive compounds like hypericin, hypocrellin, and porphyrin have been used to lyse enveloped viruses since the last century. Hypericin is active against many enveloped viruses (33) and is also cytotoxic (65, 66). Further, as expected from its mechanism of action, hypericin toxicity increases with light exposure (65, 66), as does its antiviral potency (5 to 300-fold difference between light and dark treatments for equine infectious anemia virus, murine cytomegalovirus, Sindbis virus, HIV-1, and HSV-1 [67–71]). Hypericin also inhibits antioxidant enzymes independently of light exposure, thus potentiating any direct effect (71). Therefore, hypericin is not likely to be a suitable structure from which to develop antiviral compounds.

Porphyrins act mainly as type II photosensitizers, reacting with oxygen and leading to singlet oxygen via energy transfer (72). They damage lipids, proteins, and nucleic acids, as expected for a type II photosensitizer (34). Their potency and targets depend on the chemical structure, dose, and virus (37, 73). Considering their mechanism of action, it is not surprising that these molecules inactivate enveloped as well as nonenveloped viruses.

Rhodamine (74) and thiobarbituric derivatives (type II photosensitizers) also display antiviral activity against seven enveloped viruses, and these were also proposed to act as peroxidators (36). All of these compounds have photoactive reactive groups that can start peroxidation chain reactions, as does LJ001, a lipophilic thiazolidine derivative with an SI of 66 to 100 (39). LJ001 does not have any major effect on viral proteins but inhibits fusion between virions and cellular membranes (39). It induces peroxidation in model liposomes and in free lipids, and virion peroxidation has not been evaluated. Its antiviral activity requires light, and this result is consistent with a light-activated peroxidation model. LJ001 derivatives with antiviral activity generate singlet oxygen (40) in a DMA solution (treated with 18,000 mJ/cm^2^) and in Si-DMA labeled cells irradiated at 640 nm for 1 h and evaluated in real-time by fluorescence microscopy. The antiviral potency was evaluated against PRV-GFP in PK-15 cells under different conditions (40).

The RAFIs are another group of lipophilic compounds that specifically target the infectivity of enveloped virions with little cytotoxicity (27). However, they have no major preference for intercalating into the virion envelope over cellular membranes or for obvious reactive groups (75). The RAFIs distribute through all cellular membranes as well as virion envelopes, and they affect infectivity but not cell viability (38, 75). They inhibit the infectivity of many otherwise unrelated enveloped viruses with SI > 200 (CC_50_ was not reached at any soluble concentration), but not that of nonenveloped viruses (38). They inhibit the fusion of the outer leaflets of the viral envelope to the cellular membranes before the lipids can mix. They also inhibit the formation of negative curvature by protein-free multilamellar lipid bilayers, as evaluated by isothermic calorimetry (38). Contrary to what would be expected from lipoperoxidation, the RAFIs do not disrupt virion envelopes, as evaluated by the full complement of glycoproteins, tegument, capsid proteins, and DNA in treated virions. Moreover, they do not disrupt protein-free multilamellar lipid bilayers, with the phase transitions being reversible through many iterations (27, 38). The perylene in some active RAFIs was proposed to be a photosensitizing moiety (50), but perylene is neither required nor sufficient for antiviral activity (75). Likewise, light absorption was proposed to be required to activate the antiviral activity of the RAFIs (50), but some active RAFIs do not absorb much light in the visible spectrum (75), and at least one RAFI, aUY11, is not inactivated, even after many months of exposure to light at room temperature. The mechanism of action of the RAFIs is still not totally clear, but their activity depends on the depth of insertion of the hydrophobic moiety into the outer leaflet of the viral envelope, which must be at least 10 Å, and on the energy required to fuse viral envelopes with cellular membranes (75). It has been proposed that the RAFIs act as a rigid molecular blade (75). The rigid and planar lipophilic moiety inserted into the outer leaflet would impede the lipid rearrangements and the tail bending required to avoid the energetically prohibited voids that would otherwise be created during the formation of the hemifusion stalk (75). On the other hand, aUY11 is about 100-fold less potent when it is incubated with virions in the dark and the infected cells are kept in total darkness, although it is fully active in semitransparent plastic tubes and in medium containing phenol red when the infected cells are incubated in the normal darkness of a CO_2_ incubator. The bases for this light effect on the potency of aUY11 remain mostly unclear.

The preferential sensitivity of virion envelopes over cellular membranes to peroxidative damage was proposed based on the effects of a compound already known to be virion specific, LJ001 (50), and it was also proposed as the antiviral mechanism for the RAFIs (50), which were previously demonstrated to affect virions but not cells (50, 76). The peroxidative activity of some of these compounds had been tested on lipids and on protein-free liposomes but not on actual virions (49). The model had not been tested with well-characterized, general, non-virion specific lipoperoxidators, either, although it predicts these compounds to affect virions preferentially over cells. This model was built on the presumed difference that whereas cells have a battery of approaches by which to cope with membrane peroxidation, such as catalase, superoxide dismutase, or glutathione peroxidase, and remodel membranes, virions would have no such mechanisms by which to prevent or counteract membrane peroxidation (23). However, virion envelopes are as protein rich as the inner mitochondrial membrane, which is highly resistant to oxidative insult and is particularly rich in proteins within disulfide bridges and other cysteine residues. Also, they are not enriched over cellular membranes in unsaturated lipids, which would favor the lipoperoxidation chain reaction. The high protein content in the virion envelope could well block the chain reaction of lipid peroxidation (77). This alternative model would predict that virions would not be particularly sensitive to general lipoperoxidators, which is consistent with the results obtained here with two well-characterized general lipid peroxidators.

In summary, virions are not particularly sensitive to lipoperoxidators, and antioxidants did not protect much against virion lipoperoxidation, indicating that the virion envelope is not particularly prone or sensitive to lipoperoxidative damage. Moreover, lipid peroxidators also affected infectivity independently of lipoperoxidation, likely by inducing peroxidative damage to other virion components, such as proteins or nucleic acids, and their effects are quenched by serum albumin. Thus, general lipid peroxidators have no specific antiviral activity, inhibit infectivity by mechanisms other than lipoperoxidation, and are not likely to be potent in the presence of serum. General lipoperoxidators could thus be used as virucides, but in order to develop them as antivirals, it would be necessary to develop lipoperoxidators that specifically target the virion envelope while sparing all cellular membranes. Moreover, compounds that inhibit viral replication without affecting cell viability and fail to discriminate between cellular and viral membranes are unlikely to act primarily by inducing lipoperoxidation.

## MATERIALS AND METHODS

### Compounds.

2,2′-azobis(2,4-dimethylvaleronitrile) (AMVN, Matrix Scientific, 097959) was stored at −20°C as 50 mM DMSO stock solutions (Sigma). 2,2′-azobis(2-methylpropionamidine) dihydrochloride (AAPH, Sigma-Aldrich, 440914) was stored in aliquoted pellets at −20°C.

100 mM stocks of alpha tocopherol, referred to as vitamin E, or l-ascorbic acid, referred to as vitamin C (Sigma, T3251 and AA44030), were dissolved in absolute ethanol or DMEM, respectively, and then aliquoted and stored at −20°C. Each aliquot was thawed no more than 5 times.

10 percent bovine serum albumin (BSA) stocks were prepared in milliQ water, filtered, aliquoted to be used only once, and stored at −20°C. 100 mM cholesterol (Sigma, C8667) stocks were prepared in ethanol, aliquoted, and stored at −20°C.

Lipid peroxidation was probed with C11-Bodipy (Invitrogen 581/591, D3861) and liperfluo (Dojindo, L248). C11-Bodipy stocks were dissolved in DMSO at 1 mM, aliquoted to be used no more than 3 times, and stored at −20°C. Liperfluo stocks were dissolved at 1 mM in DMSO 30 min before use. Working stocks of both probes were prepared in DMEM (Gibco) immediately before use.

### Cells and virus.

Vero African green monkey kidney cells and HFF human foreskin fibroblasts (ATCC, CCL-81, and SCRC-1041, p 12 to 30) were maintained in DMEM supplemented with 5% fetal bovine serum at 37°C in 5% CO_2_.

HSV-1 KOS (p 3 or 4 from ATCC, VR-1493) stock was prepared by infecting Vero cells at a multiplicity of infection (MOI) of 0.01 PFU/cell for 1 h at 37°C. The inocula were then removed, and the cells were washed twice with cold DMEM. Fresh prewarmed DMEM with 5% FBS was added to the infected cells, which were then incubated at 37°C in 5% CO_2_ and harvested when full CPE was visible (typically, 3 to 4 days after infection). The viral stocks were harvested in DMEM with no FBS.

### Evaluation of the effects of the lipoperoxidators on cell viability.

To evaluate the effects of AMVN or AAPH on cell viability, 7,500 HFF or Vero cells were seeded into each well of 96-well plates and incubated at 37°C in 5% CO_2_ for 18 h. Twofold serial dilutions of AMVN or AAPH in DMEM were prepared just before use and were transferred into a 96-well plate, which also included vehicle controls. The cells were washed twice with warm DMEM before a 100 μL lipoperoxidator solution was transferred with a multichannel pipette from the plate containing the drug dilutions in technical triplicates. The cells were incubated with peroxidators for 2 h at 37°C. Then, the medium was removed, and the cells were washed twice with DMEM at 37°C. The plates were cooled down to RT, and the medium was removed. Then, 50 μL phenol red free DMEM and 50 μL CellTiter-glo Luminiscent Cell Viability Assay (Promega, G7571/2/3) were added and mixed in an orbital shaker for 2 min. Afterwards, 80 μL of the mix was transferred onto white 96-well plates and incubated for 10 min at RT for signal stabilization. The luminescence was read in the Glomax Multidetection System (Promega) with an integration time of 0.5 s.

The means of the technical triplicates were normalized to the means of the vehicle controls, analyzed via nonlinear regression dose responses, and graphed using GraphPad Prism 9.0. Three or four independent biological replicates were performed for each treatment (*n* = 3 or 4).

### Evaluation of the effects of the lipoperoxidators on virion infectivity.

To evaluate the effects of AMVN or AAPH on virion infectivity, 1.5 × 10^5^ HFF or Vero cells were seeded into each well of 12-well plates and incubated at 37°C in 5% CO_2_ for 18 h. HSV-1 suspensions of 20,000 plaque PFU in 250 μL DMEM were mixed 1:1 with 2-fold serial dilutions of the general peroxidators and were incubated in a water bath for 2 h at 37°C. Next, treated virions were diluted 1:100 before being adsorbed onto cells for 1 h at 37°C. The inocula were removed, and the cells were washed twice with cold DMEM. Infected cells were overlaid with DMEM supplemented with 2% (Vero) or 1% (HFF) methylcellulose and 5% FBS and were incubated at 37°C until plaques were visible and well-resolved, typically in about 2 days. Cell monolayers were fixed and stained with 1% crystal violet in 17% methanol for 18 h at RT. The cells were washed, and the plaques were counted and normalized to those produced by the vehicle treated virions.

To evaluate any effect on the cell viability of AMVN preloaded into viral envelopes, 5 × 10^3^ PFU of HSV-1 were incubated 1:1 with serial dilutions of AMVN for 2 h at 37°C before being diluted 1:100 and added to cells. After 1 h at 37°C, the cells were washed, and cell viability was evaluated as described. An aliquot of the diluted treated virions were tested for infectivity, and the aliquots of each AMVN dilution were tested for direct effects on cell viability.

### Evaluation of the effects of antioxidants on cell viability and virion infectivity.

To evaluate the effects of lipid soluble antioxidants, 10 μM vitamin E was loaded onto carrier 1% BSA in the dark for 4.5 h at RT. The cells were washed twice with DMEM at 37°C and preloaded with 100 μL of 10 μM vitamin E in BSA carrier for 3 h at 37°C. The cells were washed twice again before the addition of any lipoperoxidators. Peroxidators were then added to the cells at working concentrations and incubated for 2 h at 37°C. The peroxidation solution was then removed, and the cells were washed twice with DMEM before analyzing viability. To evaluate the effects of a water soluble antioxidant, the cells were treated with 10 μM vitamin C (final concentration) simultaneously with the lipoperoxidators.

To test the effects of lipid soluble antioxidants on virions, 10 μM vitamin E loaded onto 1% carrier BSA in the dark for 4.5 h at RT was incubated with 10^6^ HSV-1 virions for 3 h at 37°C. AMVN or AAPH was then added and incubated in a water bath for 2 h at 37°C. The treated virions were diluted 1:100 before infecting cells. To test the effect of water soluble antioxidants, HSV-1 suspensions (5 × 10^5^ PFU in 250 μL) in a 10 μM vitamin C solution were treated with AMVN or AAPH. The mix was incubated for 2 h at 37°C, diluted 1:100, and used to infect cells. The infectivity of an aliquot of treated virions was tested via a plaque assay.

### Evaluation of lipid peroxidation.

To evaluate cell lipid peroxidation using liperfluo, 7.5 × 10^3^ Vero cells were seeded into each well of 96-well plates and incubated for 18 h at 37°C in 5% CO_2_. Cells were then preloaded with 20 μM liperfluo for 30 min at 37°C before adding AMVN or AAPH dilutions in technical triplicates. Two hours later, the cells were washed twice, and the fluorescence was evaluated in a TECAN Infinite M200 Pro at 515 nm excitation and 545 nm emission. The results are presented relative to vehicle-treated cells.

To evaluate virion peroxidation using liperfluo, 50 μL DMEM that contained 5 × 10^6^ PFU of HSV-1 was added to 96-well plates. Virions were then preloaded with 10 μM liperfluo for 20 min at 37°C. Next, AMVN or AAPH dilutions were added and incubated for 2 h at 37°C. Fluorescence was evaluated in a TECAN Infinite M200 Pro at 515 nm excitation and 545 nm emission. Medium containing no virus was used as blank. The results are presented relative to vehicle treated virions.

To evaluate Vero cell lipoperoxidation with C11-Bodipy, 7.5 × 10^3^ cells were seeded into each well of 96-well plates and incubated for 18 h at 37°C in 5% CO_2_ before preloading them with 1 μM C11-Bodipy for 30 min at 37°C. Next, AMVN or AAPH dilutions were added in technical triplicates. Two hours later, the cells were washed twice and lysed with DMSO:isopropanol. Fluorescence was evaluated in a TECAN Infinite M200 Pro at 550 nm excitation and 580 to 630 nm emission with the reading peak at 600 nm. The results are presented relative to vehicle treated cells.

To evaluate virion lipoperoxidation with C11-Bodipy, 50 μL DMEM containing 5 × 10^6^ PFU of HSV-1 was added into each well of 96-well plates. Virions were then preloaded with 0.1 mol % of C11-Bodipy for 30 min at 37°C. Next, AMVN or AAPH dilutions were added and incubated for 2 h at 37°C. Fluorescence was evaluated in a TECAN Infinite M200 Pro at 550 nm excitation and 580 to 630 nm emission with the reading peak at 600 nm. The results are presented relative to vehicle treated cells.

### Antioxidant effects on lipid peroxidation.

To evaluate the effects of a lipid soluble antioxidant, cells or virions were preloaded with vitamin E-BSA for 3 h at 37°C. Vitamin E-BSA containing medium was removed, and the cells were washed twice with DMEM. Liperfluo (20 or 10 μM for cells or virions, respectively) or C11-Bodipy (1 μM for cells or 0.1 mol% for virions) were preloaded into the cells or virions for 30 min at 37°C. Then, AMVN or AAPH was added and incubated for 2 h at 37°C.

To evaluate the effects of water soluble antioxidants, liperfluo (20 μM for cells or 10 μM for virions) or C11-Bodipy (1 μM for cells or 0.1 mol% for virions) were preloaded into the cells or virions for 30 min at 37°C. Next, the cells or virions were treated with a 10 μM vitamin C solution and AMVN or AAPH and then incubated for 2 h at 37°C. Fluorescence was evaluated in a TECAN Infinite M200 Pro at 515 nm excitation and 545 nm emission for liperfluo or at 550 nm excitation and 580 to 630 nm emission for C11-Bodipy. The results are presented relative to vehicle treated cells.

### Atmospheric oxidative conditions.

A time course plaquing efficiency assay was performed with HSV-1 incubated under atmospheric oxidative conditions. Virions (3 × 10^5^ PFU) were incubated with vitamin E, vitamin E-BSA, cholesterol, cholesterol-BSA, vitamin C (10 μM final concentration), or the carrier BSA (0.5% final concentration) for 3 h at 37°C in a water bath with atmospheric air (atmospheric O_2_). Aliquots from each condition were taken every hour and diluted 1:200 to perform plaquing efficiency assays as previously described on the Vero cells.

### Lipid peroxidation under atmospheric oxidative conditions.

To evaluate virion peroxidation using liperfluo, 250 μL DMEM that contained 5 × 10^5^ PFU of HSV-1 incubated under atmospheric conditions were preloaded with 10 μM liperfluo for 30 min at 37°C (at 0, 30, 90 and 160 min). The virions preloaded with the probe were transferred to a 96 well-plate at 30 min as well as at 1, 2, and 3 h. Fluorescence was evaluated in a TECAN Infinite M200 Pro at 515 nm excitation and 545 nm emission.

To evaluate virion lipoperoxidation with C11-Bodipy, 250 μL DMEM containing 5 × 10^5^ PFU HSV-1 were preloaded with 0.1 mol % of C11-Bodipy for 30 min at 37°C (at 0, 30, 90 and 160 min). The virions preloaded with the probe were transferred to a 96 well-plate at 30 min as well as at 1, 2, and 3 h. Fluorescence was evaluated in a TECAN Infinite M200 Pro at 550 nm excitation and 580 to 630 nm emission with the reading peak at 600 nm. The results are presented relative to vehicle treated cells.

## References

[1] FDA. 2022. Novel drug approvals for 2022, on U.S. Food and Drug Administration. https://www.fda.gov/drugs/new-drugs-fda-cders-new-molecular-entities-and-new-therapeutic-biological-products/novel-drug-approvals-2022. Accessed July 5 2022.

[2] Tompa DR, Immanuel A, Srikanth S, Kadhirvel S. 2021. Trends and strategies to combat viral infections: a review on FDA approved antiviral drugs. Int J Biol Macromol 172:524–541. doi:10.1016/j.ijbiomac.2021.01.076. 33454328 PMC8055758

[3] De Clercq E, Li G. 2016. Approved antiviral drugs over the past 50 years. Clin Microbiol Rev 29:695–747. doi:10.1128/CMR.00102-15. 27281742 PMC4978613

[4] Pisaturo M, Onorato L, Russo A, Martini S, Chiodini P, Signoriello S, Maggi P, Coppola N. 2021. Risk of failure in dual therapy versus triple therapy in naive HIV patients: a systematic review and meta-analysis. Clin Microbiol Infect 27:28–35. doi:10.1016/j.cmi.2020.09.048. 33031949

[5] Wright P, Kelsall J, Healing G, Sanderson J. 2019. Differential expression of cyclin-dependent kinases in the adult human retina in relation to CDK inhibitor retinotoxicity. Arch Toxicol 93:659–671. doi:10.1007/s00204-018-2376-8. 30617560

[6] Zhang M, Zhang L, Hei R, Li X, Cai H, Wu X, Zheng Q, Cai C. 2021. CDK inhibitors in cancer therapy, an overview of recent development. Am J Cancer Res 11:1913–1935. 34094661 PMC8167670

[7] Ajiro M, Sakai H, Onogi H, Yamamoto M, Sumi E, Sawada T, Nomura T, Kabashima K, Hosoya T, Hagiwara M. 2018. CDK9 inhibitor FIT-039 suppresses viral oncogenes E6 and E7 and has a therapeutic effect on HPV-induced neoplasia. Clin Cancer Res 24:4518–4528. doi:10.1158/1078-0432.CCR-17-3119. 29712686

[8] Łukasik P, Baranowska-Bosiacka I, Kulczycka K, Gutowska I. 2021. Inhibitors of cyclin-dependent kinases: types and their mechanism of action. Int J Mol Sci 22:2806. doi:10.3390/ijms22062806. 33802080 PMC8001317

[9] Schor S, Einav S. 2018. Repurposing of kinase inhibitors as broad-spectrum antiviral drugs. DNA Cell Biol 37:63–69. doi:10.1089/dna.2017.4033. 29148875 PMC5804095

[10] Kannaiyan R, Mahadevan D. 2018. A comprehensive review of protein kinase inhibitors for cancer therapy. Expert Rev Anticancer Ther 18:1249–1270. doi:10.1080/14737140.2018.1527688. 30259761 PMC6322661

[11] Thomson RJ, Moshirfar M, Ronquillo Y. 2022. Tyrosine Kinase Inhibitors, StatPearls, Treasure Island (FL). 33090752

[12] Pathania S, Rawal RK, Singh PK. 2022. RdRp (RNA-dependent RNA polymerase): a key target providing anti-virals for the management of various viral diseases. J Mol Struct 1250:131756. doi:10.1016/j.molstruc.2021.131756. 34690363 PMC8520695

[13] Elion GB. 1982. Mechanism of action and selectivity of acyclovir. Am J Med 73:7–13. doi:10.1016/0002-9343(82)90055-9. 6285736

[14] Oberg B. 1989. Antiviral effects of phosphonoformate (Pfa, foscarnet sodium). Pharmacol Ther 40:213–285. doi:10.1016/0163-7258(89)90097-1. 2543994

[15] Lee HW, Lee JS, Ahn SH. 2020. Hepatitis B virus cure: targets and future therapies. Int J Mol Sci 22. 10.3390/ijms22010213 PMC7795643 33379331

[16] Yuen MF, Gane EJ, Kim DJ, Weilert F, Yuen Chan HL, Lalezari J, Hwang SG, Nguyen T, Flores O, Hartman G, Liaw S, Lenz O, Kakuda TN, Talloen W, Schwabe C, Klumpp K, Brown N. 2019. Antiviral activity, safety, and pharmacokinetics of capsid assembly modulator NVR 3-778 in patients with chronic HBV infection. Gastroenterology 156:1392–1403e7. doi:10.1053/j.gastro.2018.12.023. 30625297

[17] Fung S, Choi HSJ, Gehring A, Janssen HLA. 2022. Getting to HBV cure: the promising paths forward. Hepatology 76:233–250. doi:10.1002/hep.32314. 34990029

[18] Bester SM, Wei G, Zhao H, Adu-Ampratwum D, Iqbal N, Courouble VV, Francis AC, Annamalai AS, Singh PK, Shkriabai N, Van Blerkom P, Morrison J, Poeschla EM, Engelman AN, Melikyan GB, Griffin PR, Fuchs JR, Asturias FJ, Kvaratskhelia M. 2020. Structural and mechanistic bases for a potent HIV-1 capsid inhibitor. Science 370:360–364. doi:10.1126/science.abb4808. 33060363 PMC7831379

[19] Anonymous. 2022. Lenacapavir FDA approval status. https://www.drugs.com/history/lenacapavir.html. Accessed March 13 2022.

[20] Carnes SK, Sheehan JH, Aiken C. 2018. Inhibitors of the HIV-1 capsid, a target of opportunity. Curr Opin HIV AIDS 13:359–365. doi:10.1097/COH.0000000000000472. 29782334 PMC6075716

[21] Egorova A, Ekins S, Schmidtke M, Makarov V. 2019. Back to the future: advances in development of broad-spectrum capsid-binding inhibitors of enteroviruses. Eur J Med Chem 178:606–622. doi:10.1016/j.ejmech.2019.06.008. 31226653 PMC8194503

[22] Chen M, Aoki-Utsubo C, Kameoka M, Deng L, Terada Y, Kamitani W, Sato K, Koyanagi Y, Hijikata M, Shindo K, Noda T, Kohara M, Hotta H. 2017. Broad-spectrum antiviral agents: secreted phospholipase A2 targets viral envelope lipid bilayers derived from the endoplasmic reticulum membrane. Sci Rep 7:15931. doi:10.1038/s41598-017-16130-w. 29162867 PMC5698466

[23] Wolf MC, Freiberg AN, Zhang T, Akyol-Ataman Z, Grock A, Hong PW, Li J, Watson NF, Fang AQ, Aguilar HC, Porotto M, Honko AN, Damoiseaux R, Miller JP, Woodson SE, Chantasirivisal S, Fontanes V, Negrete OA, Krogstad P, Dasgupta A, Moscona A, Hensley LE, Whelan SP, Faull KF, Holbrook MR, Jung ME, Lee B. 2010. A broad-spectrum antiviral targeting entry of enveloped viruses. Proc Natl Acad Sci USA 107:3157–3162. doi:10.1073/pnas.0909587107. 20133606 PMC2840368

[24] Nieto-Garai JA, Glass B, Bunn C, Giese M, Jennings G, Brankatschk B, Agarwal S, Borner K, Contreras FX, Knolker HJ, Zankl C, Simons K, Schroeder C, Lorizate M, Krausslich HG. 2018. Lipidomimetic compounds act as HIV-1 entry inhibitors by altering viral membrane structure. Front Immunol 9:1983. doi:10.3389/fimmu.2018.01983. 30233582 PMC6131562

[25] Bukrinsky MI, Mukhamedova N, Sviridov D. 2020. Lipid rafts and pathogens: the art of deception and exploitation. J Lipid Res 61:601–610. doi:10.1194/jlr.TR119000391. 31615838 PMC7193957

[26] Welsch S, Muller B, Krausslich HG. 2007. More than one door - budding of enveloped viruses through cellular membranes. FEBS Lett 581:2089–2097. doi:10.1016/j.febslet.2007.03.060. 17434167 PMC7126970

[27] StVincent MRM. 2012. The Discovery and Characterization of Rigid Amphipathic Fusion Inhibitors (RAFIs), a Novel Class of Broad-Spectrum Antiviral Compounds. Doctor of Philosophy. University of Alberta, Edmonton, Alberta.

[28] Rey FA, Lok SM. 2018. Common features of enveloped viruses and implications for immunogen design for next-generation vaccines. Cell 172:1319–1334. doi:10.1016/j.cell.2018.02.054. 29522750 PMC7112304

[29] Harrison SC. 2015. Viral membrane fusion. Virology 479–480:498–507. doi:10.1016/j.virol.2015.03.043. PMC4424100 25866377

[30] Chernomordik LV, Kozlov MM. 2003. Protein-lipid interplay in fusion and fission of biological membranes. Annu Rev Biochem 72:175–207. doi:10.1146/annurev.biochem.72.121801.161504. 14527322

[31] Jelesarov I, Lu M. 2001. Thermodynamics of trimer-of-hairpins formation by the SIV gp41 envelope protein. J Mol Biol 307:637–656. doi:10.1006/jmbi.2001.4469. 11254387

[32] Diwu Z. 1995. Novel therapeutic and diagnostic applications of hypocrellins and hypericins. Photochem Photobiol 61:529–539. doi:10.1111/j.1751-1097.1995.tb09903.x. 7568399

[33] Mariewskaya KA, Tyurin AP, Chistov AA, Korshun VA, Alferova VA, Ustinov AV. 2021. Photosensitizing antivirals. Molecules 26:3971. doi:10.3390/molecules26133971. 34209713 PMC8271894

[34] Baptista MS, Cadet J, Greer A, Thomas AH. 2021. Photosensitization reactions of biomolecules: definition, targets and mechanisms. Photochem Photobiol 97:1456–1483. doi:10.1111/php.13470. 34133762

[35] Mousavi SM, Zarei M, Hashemi SA, Babapoor A, Amani AM. 2019. A conceptual review of rhodanine: current applications of antiviral drugs, anticancer and antimicrobial activities. Artif Cells Nanomed Biotechnol 47:1132–1148. doi:10.1080/21691401.2019.1573824. 30942110

[36] Cagno V, Tintori C, Civra A, Cavalli R, Tiberi M, Botta L, Brai A, Poli G, Tapparel C, Lembo D, Botta M. 2018. Novel broad spectrum virucidal molecules against enveloped viruses. PLoS One 13:e0208333. doi:10.1371/journal.pone.0208333. 30532192 PMC6285983

[37] Lebedeva NS, Gubarev YA, Koifman MO, Koifman OI. 2020. The application of porphyrins and their analogues for inactivation of viruses. Molecules 25:4368. doi:10.3390/molecules25194368. PMC7583985 32977525

[38] Colpitts CC, Ustinov AV, Epand RF, Epand RM, Korshun VA, Schang LM. 2013. 5-(Perylen-3-yl) ethynyl-arabino-uridine (aUY11), an arabino-based rigid amphipathic fusion inhibitor, targets virion envelope lipids to inhibit fusion of influenza virus, hepatitis C virus, and other enveloped viruses. J Virol 87:3640–3654. doi:10.1128/JVI.02882-12. 23283943 PMC3624206

[39] Vigant F, Lee J, Hollmann A, Tanner LB, Akyol Ataman Z, Yun T, Shui G, Aguilar HC, Zhang D, Meriwether D, Roman-Sosa G, Robinson LR, Juelich TL, Buczkowski H, Chou S, Castanho MA, Wolf MC, Smith JK, Banyard A, Kielian M, Reddy S, Wenk MR, Selke M, Santos NC, Freiberg AN, Jung ME, Lee B. 2013. A mechanistic paradigm for broad-spectrum antivirals that target virus-cell fusion. PLoS Pathog 9:e1003297. doi:10.1371/journal.ppat.1003297. 23637597 PMC3630091

[40] Zeng L, Wang MD, Ming SL, Li GL, Yu PW, Qi YL, Jiang DW, Yang GY, Wang J, Chu BB. 2020. An effective inactivant based on singlet oxygen-mediated lipid oxidation implicates a new paradigm for broad-spectrum antivirals. Redox Biol 36:101601. doi:10.1016/j.redox.2020.101601. 32535542 PMC7278711

[41] Cruz-Oliveira C, Almeida AF, Freire JM, Caruso MB, Morando MA, Ferreira VNS, Assunção-Miranda I, Gomes AMO, Castanho M, Da Poian AT. 2017. Mechanisms of vesicular stomatitis virus inactivation by protoporphyrin IX, zinc-protoporphyrin IX, and mesoporphyrin IX. Antimicrob Agents Chemother 61. doi:10.1128/AAC.00053-17. PMC5444121 28348154

[42] Hirayama J, Ikebuchi K, Abe H, Kwon KW, Ohnishi Y, Horiuchi M, Shinagawa M, Ikuta K, Kamo N, Sekiguchi S. 1997. Photoinactivation of virus infectivity by hypocrellin A. Photochem Photobiol 66:697–700. doi:10.1111/j.1751-1097.1997.tb03209.x. 9383993

[43] Hollmann A, Gonçalves S, Augusto MT, Castanho MA, Lee B, Santos NC. 2015. Effects of singlet oxygen generated by a broad-spectrum viral fusion inhibitor on membrane nanoarchitecture. Nanomedicine (Lond) 11:1163–1167. doi:10.1016/j.nano.2015.02.014. PMC4476930 25791807

[44] Yin HY, Xu LB, Porter NA. 2011. Free radical lipid peroxidation: mechanisms and analysis. Chem Rev 111:5944–5972. doi:10.1021/cr200084z. 21861450

[45] Ayala A, Munoz MF, Arguelles S. 2014. Lipid peroxidation: production, metabolism, and signaling mechanisms of malondialdehyde and 4-hydroxy-2-nonenal. Oxid Med Cell Longev 2014:360438. doi:10.1155/2014/360438. 24999379 PMC4066722

[46] Yadav DK, Kumar S, Choi EH, Chaudhary S, Kim MH. 2019. Molecular dynamic simulations of oxidized skin lipid bilayer and permeability of reactive oxygen species. Sci Rep 9:4496. doi:10.1038/s41598-019-40913-y. 30872693 PMC6418262

[47] Del Rio D, Stewart AJ, Pellegrini N. 2005. A review of recent studies on malondialdehyde as toxic molecule and biological marker of oxidative stress. Nutr Metab Cardiovasc Dis 15:316–328. doi:10.1016/j.numecd.2005.05.003. 16054557

[48] Schaur RJ. 2003. Basic aspects of the biochemical reactivity of 4-hydroxynonenal. Mol Aspects Med 24:149–159. doi:10.1016/s0098-2997(03)00009-8. 12892992

[49] Hollmann A, Castanho MA, Lee B, Santos NC. 2014. Singlet oxygen effects on lipid membranes: implications for the mechanism of action of broad-spectrum viral fusion inhibitors. Biochem J 459:161–170. doi:10.1042/BJ20131058. 24456301

[50] Vigant F, Hollmann A, Lee J, Santos NC, Jung ME, Lee B. 2014. The rigid amphipathic fusion inhibitor dUY11 acts through photosensitization of viruses. J Virol 88:1849–1853. doi:10.1128/JVI.02907-13. 24284320 PMC3911596

[51] Lafin JT, Sarsour EH, Kalen AL, Wagner BA, Buettner GR, Goswami PC. 2019. Methylseleninic acid induces lipid peroxidation and radiation sensitivity in head and neck cancer cells. Int J Mol Sci 20. doi:10.3390/ijms20010225. PMC6337472 30626124

[52] Sunjic SB, Gasparovic AC, Jaganjac M, Rechberger G, Meinitzer A, Grune T, Kohlwein SD, Mihaljevic B, Zarkovic N. 2021. Sensitivity of osteosarcoma cells to concentration-dependent bioactivities of lipid peroxidation product 4-hydroxynonenal depend on their level of differentiation. Cells 10 10:269. doi:10.3390/cells10020269. PMC7912392 33572933

[53] Mosca M, Ceglie A, Ambrosone L. 2011. Effect of membrane composition on lipid oxidation in liposomes. Chem Phys Lipids 164:158–165. doi:10.1016/j.chemphyslip.2010.12.006. 21185813

[54] Chatterjee SN, Agarwal S. 1988. Liposomes as membrane model for study of lipid peroxidation. Free Radic Biol Med 4:51–72. doi:10.1016/0891-5849(88)90011-1. 2830175

[55] Schnitzer E, Pinchuk I, Bor A, Leikin-Frenkel A, Lichtenberg D. 2007. Oxidation of liposomal cholesterol and its effect on phospholipid peroxidation. Chem Phys Lipids 146:43–53. doi:10.1016/j.chemphyslip.2006.12.003. 17241622

[56] Niki E. 1990. Free-radical initiators as source of water-soluble or lipid-soluble peroxyl radicals. Methods Enzymol 186:100–108. doi:10.1016/0076-6879(90)86095-d. 2233287

[57] Krainev AG, Bigelow DJ. 1996. Comparison of 2,2'-azobis(2-amidinopropane) hydrochloride (AAPH) and 2,2'azobis(2,4-dimethylvaleronitrile) (AMVN) as free radical initiators: a spin-trapping study. J the Chemical Society-Perkin Transactions 2:747–754. doi:10.1039/p29960000747.

[58] Shiva Shankar Reddy CS, Subramanyam MV, Vani R, Asha Devi S. 2007. In vitro models of oxidative stress in rat erythrocytes: effect of antioxidant supplements. Toxicol in Vitro 21:1355–1364. doi:10.1016/j.tiv.2007.06.010. 17714909

[59] Yan J, Gong Y, She YM, Wang G, Roberts MS, Burczynski FJ. 2009. Molecular mechanism of recombinant liver fatty acid binding protein's antioxidant activity. J Lipid Res 50:2445–2454. doi:10.1194/jlr.M900177-JLR200. 19474456 PMC2781316

[60] Takenaka Y, Miki M, Yasuda H, Mino M. 1991. The effect of alpha-tocopherol as an antioxidant on the oxidation of membrane protein thiols induced by free radicals generated in different sites. Arch Biochem Biophys 285:344–350. doi:10.1016/0003-9861(91)90370-x. 1897937

[61] Schnitzer E, Pinchuk I, Lichtenberg D. 2007. Peroxidation of liposomal lipids. Eur Biophys J 36:499–515. doi:10.1007/s00249-007-0146-2. 17380326

[62] Pinchuk I, Lichtenberg D. 2014. Analysis of the kinetics of lipid peroxidation in terms of characteristic time-points. Chem Phys Lipids 178:63–76. doi:10.1016/j.chemphyslip.2013.12.001. 24333462

[63] Qin T, Ma R, Yin Y, Miao X, Chen S, Fan K, Xi J, Liu Q, Gu Y, Yin Y, Hu J, Liu X, Peng D, Gao L. 2019. Catalytic inactivation of influenza virus by iron oxide nanozyme. Theranostics 9:6920–6935. doi:10.7150/thno.35826. 31660077 PMC6815955

[64] Murray BK, Ohmine S, Tomer DP, Jensen KJ, Johnson FB, Kirsi JJ, Robison RA, O'Neill KL. 2008. Virion disruption by ozone-mediated reactive oxygen species. J Virol Methods 153:74–77. doi:10.1016/j.jviromet.2008.06.004. 18598719

[65] Schmitt LA, Liu Y, Murphy PA, Birt DF. 2006. Evaluation of the light-sensitive cytotoxicity of Hypericum perforatum extracts, fractions, and pure compounds. J Agric Food Chem 54:2881–2890. doi:10.1021/jf052344k. 16608204 PMC1557644

[66] Mirmalek SA, Azizi MA, Jangholi E, Yadollah-Damavandi S, Javidi MA, Parsa Y, Parsa T, Salimi-Tabatabaee SA, Ghasemzadeh Kolagar H, Alizadeh-Navaei R. 2015. Cytotoxic and apoptogenic effect of hypericin, the bioactive component of Hypericum perforatum on the MCF-7 human breast cancer cell line. Cancer Cell Int 16:3. doi:10.1186/s12935-016-0279-4. 26865836 PMC4748624

[67] Carpenter S, Kraus GA. 1991. Photosensitization is required for inactivation of equine infectious-anemia virus by hypericin. Photochem Photobiol 53:169–174. doi:10.1111/j.1751-1097.1991.tb03919.x. 1707176

[68] Hudson JB, Lopez-Bazzocchi I, Towers GH. 1991. Antiviral activities of hypericin. Antiviral Res 15:101–112. doi:10.1016/0166-3542(91)90028-p. 1650164

[69] Cohen PA, Hudson JB, Towers GH. 1996. Antiviral activities of anthraquinones, bianthrones and hypericin derivatives from lichens. Experientia 52:180–183. doi:10.1007/BF01923366. 8608821

[70] Birt DF, Widrlechner MP, Hammer KD, Hillwig ML, Wei J, Kraus GA, Murphy PA, McCoy J, Wurtele ES, Neighbors JD, Wiemer DF, Maury WJ, Price JP. 2009. Hypericum in infection: identification of anti-viral and anti-inflammatory constituents. Pharm Biol 47:774–782. doi:10.1080/13880200902988645. 19907671 PMC2774925

[71] Kubin A, Wierrani F, Burner U, Alth G, Grunberger W. 2005. Hypericin - the facts about a controversial agent. Curr Pharm Des 11:233–253. doi:10.2174/1381612053382287. 15638760

[72] Foote CS. 1991. Definition of type I and type II photosensitized oxidation. Photochem Photobiol 54:659–659. doi:10.1111/j.1751-1097.1991.tb02071.x. 1798741

[73] Korneev D, Kurskaya O, Sharshov K, Eastwood J, Strakhovskaya M. 2019. Ultrastructural aspects of photodynamic inactivation of highly pathogenic avian H5N8 influenza virus. Viruses 11:955. doi:10.3390/v11100955. PMC6832225 31623281

[74] Liu C, Zhou L, Wei F, Li L, Zhao S, Gong P, Cai L, Wong KM. 2019. Versatile strategy to generate a rhodamine triplet state as mitochondria-targeting visible-light photosensitizers for efficient photodynamic therapy. ACS Appl Mater Interfaces 11:8797–8806. doi:10.1021/acsami.8b20224. 30730131

[75] Speerstra S, Chistov AA, Proskurin GV, Aralov AV, Ulashchik EA, Streshnev PP, Shmanai VV, Korshun VA, Schang LM. 2018. Antivirals acting on viral envelopes via biophysical mechanisms of action. Antiviral Res 149:164–173. doi:10.1016/j.antiviral.2017.11.018. 29191427

[76] St Vincent MR, Colpitts CC, Ustinov AV, Muqadas M, Joyce MA, Barsby NL, Epand RF, Epand RM, Khramyshev SA, Valueva OA, Korshun VA, Tyrrell DLJ, Schang LM. 2010. Rigid amphipathic fusion inhibitors, small molecule antiviral compounds against enveloped viruses. Proc Natl Acad Sci USA 107:17339–17344. doi:10.1073/pnas.1010026107. 20823220 PMC2951442

[77] Tweeddale HJ, Kondo M, Gebicki JM. 2007. Proteins protect lipid membranes from oxidation by thiyl radicals. Arch Biochem Biophys 459:151–158. doi:10.1016/j.abb.2007.01.016. 17306209

